# Genetic landscape of primary mitochondrial diseases in children and adults using molecular genetics and genomic investigations of mitochondrial and nuclear genome

**DOI:** 10.1186/s13023-024-03437-x

**Published:** 2024-11-12

**Authors:** Anastasia Ambrose, Shalini Bahl, Saloni Sharma, Dan Zhang, Clara Hung, Shailly Jain-Ghai, Alicia Chan, Saadet Mercimek-Andrews

**Affiliations:** 1 Department of Medical Genetics, Faculty of Medicine and Dentistry, University of Alberta, 8-39 Medical Sciences Building, 8613 114 Street, Edmonton, AB T6G 2H7 Canada; 2 Princess Margaret Cancer Centre, 101 College Street, Toronto, ON M5G 1L7 Canada; 3 Department of Medical Biophysics, University of Toronto, 101 College Street, Toronto, ON M5G 1L7 Canada; 4 Neuroscience and Mental Health Institute, University of Alberta, Edmonton, AB Canada; 5 Women and Children’s Health Research Institute, University of Alberta, Edmonton, AB Canada; 6 Alberta Health Services, Edmonton Zone, AB Canada

**Keywords:** Primary mitochondrial diseases, Exome sequencing, Mitochondrial genome sequencing

## Abstract

**Background:**

Primary mitochondrial diseases (PMD) are one of the most common metabolic genetic disorders. They are due to pathogenic variants in the mitochondrial genome (mtDNA) or nuclear genome (nDNA) that impair mitochondrial function and/or structure. We hypothesize that there is overlap between PMD and other genetic diseases that are mimicking PMD. For this reason, we performed a retrospective cohort study.

**Methods:**

All individuals with suspected PMD that underwent molecular genetic and genomic investigations were included. Individuals were grouped for comparison: (1) individuals with mtDNA-PMD; (2) individuals with nDNA-PMD; (3) individuals with other genetic diseases mimicking PMD (non-PMD); (4) individuals without a confirmed genetic diagnosis.

**Results:**

297 individuals fulfilled inclusion criteria. The diagnostic yield of molecular genetics and genomic investigations was 31.3%, including 37% for clinical exome sequencing and 15.8% for mitochondrial genome sequencing. We identified 71 individuals with PMD (mtDNA *n* = 41, nDNA *n* = 30) and 22 individuals with non-PMD. Adults had higher percentage of mtDNA-PMD compared to children (*p*-value = 0.00123). There is a statistically significant phenotypic difference between children and adults with PMD.

**Conclusion:**

We report a large cohort of individuals with PMD and the diagnostic yield of urine mitochondrial genome sequencing (16.1%). We think liver phenotype might be progressive and should be studied further in PMD. We showed a relationship between non-PMD genes and their indirect effects on mitochondrial machinery. Differentiation of PMD from non-PMD can be achieved using specific phenotypes as there was a statistically significant difference for muscular, cardiac, and ophthalmologic phenotypes, seizures, hearing loss, peripheral neuropathy in PMD group compared to non-PMD group.

**Supplementary Information:**

The online version contains supplementary material available at 10.1186/s13023-024-03437-x.

## Background

Primary mitochondrial diseases (PMD) are one of the most common inherited metabolic disorders. They are due to pathogenic variants in the mitochondrial or nuclear genome that impair mitochondrial function and energy production. The prevalence of PMD is about 1 in 4000 live births. There are more than 500 different genetic defects causing PMD [1, 2].

Almost every cell in the human body has mitochondria which are the energy production machinery of the cells. The electron transport chain, located in the inner mitochondrial membrane, is crucial for oxidative phosphorylation and ATP production in the cell [3–5]. Molecular genetic defects of mitochondrial and nuclear genome disrupt the function of the five complexes in the electron transport chain, leading to impaired mitochondrial function and reduced energy production [5–8]. Functional and structural defects of mitochondria affect multiple organs, especially the high energy requiring organs such as brain, retina, cardiac and skeletal muscle, but also liver, kidney, endocrine and gastrointestinal systems. The phenotypes of PMD are on a spectrum ranging from prenatal or neonatal onset lethal disease affecting multiple organ systems to adult onset progressive external ophthalmoplegia. The disease onset is in childhood in two-thirds of the individuals with PMD [9, 10].

Mitochondrial disease criteria were applied for the diagnostic confirmation of suspected PMD prior to 2013 and new clinical criteria were developed in the absence of molecular genetics and genomic investigations recently [11–14]. Until recently, numerous investigations were applied to help in the diagnosis of PMD including biochemical investigations (e.g. lactate, pyruvate, amino acid and organic acid analyses), exercise test, muscle biopsy, brain magnetic resonance imaging (MRI), brain magnetic resonance spectroscopy (MRS) and lumbar puncture. Additionally, individuals with suspected PMD undergo echocardiography, endocrinological investigations, ultrasounds of liver and kidney, hearing tests and ophthalmologic exam to investigate if there is any other organ involvement. In the recent years, molecular genetics and genomic investigations have been applied to confirm underlying genetic diagnosis in individuals with suspected PMD [15–19]. As the mitochondrial DNA variant load can vary between tissues, mitochondrial DNA should be extracted from the most affected organ for molecular genetics and genomic investigations. Commonly used tissues include blood, muscle, buccal swabs, and skin fibroblasts [20–22]. Despite extensive investigations, the underlying genetic defect may not be identified. This may prematurely end the individuals’ diagnostic journey, overmedicalize their care and potentially limit access to appropriate treatments for the actual underlying genetic disease [13, 23].

We hypothesize that there is an overlap between PMD and other genetic diseases that mimic PMD. For this reason, we performed a retrospective cohort study to report the: (1) Genetic landscape of PMD in Alberta; (2) Diagnostic yield of mitochondrial genetics and genomic investigations; and (3) Comparison of phenotypes, biochemical features, muscle histopathology, electron transport chain activities, neuroimaging, and neurophysiological studies between individuals with PMD and with other genetic diseases mimicking PMD (non-PMD).

## Methods

Alberta Research Information Services (ARISE) at the University of Alberta approved this study (Approval ID: Pro00112487). Northern Alberta Clinical Trials and Research Centre (NACTRC) approved this clinical research study for the use of Alberta Health Services Data (Approval ID: PRJ38205). We used different databases including FoxPro, Sunquest, Connect Care and Metabolic Genetics Clinic Databases and generated an Excel Database. Our inclusion criteria were: (1) All individuals with suspected PMD who were referred to our metabolic genetics clinic at the University of Alberta; and/or (2) All individuals who had any of the following molecular genetics and genomic investigations: targeted next generation sequencing panels, clinical exome sequencing, mitochondrial deletion/duplication testing, mitochondrial genome sequencing, and common mitochondrial variant testing.

We reviewed Electronic Patient Charts for the clinical features, biochemical investigations, cardiac assessments, neuroimaging features, and molecular genetics and genomic investigations. We entered all information into an Excel database (Microsoft Corp., Redmond, WA, U.S.A.).

Molecular genetics and genomic investigations using individual and/or parents’ DNA samples were performed in clinical molecular genetics and genomic laboratories according to their methods. American College of Medical Genetics and Genomics and the Association for Molecular Pathology (ACMG/AMP) variant classification guidelines for interpretation of genetic variants were applied [24, 25]. All variants in the Genome Aggregation Database (gnomAD v3.2.1) (http://gnomad.broadinstitute.org/about) for their allele frequency in the general population were searched [26].

We divided individuals into four groups to compare their phenotypes and genetic diagnoses including: (1) Group 1: individuals with genetically confirmed mtDNA-PMD; (2) Group 2: individuals with genetically confirmed nDNA-PMD; (3) Group 3: individuals with other genetic diseases that are mimicking PMD (non-PMD); (4) Group 4: individuals with no confirmed genetic diagnosis. We analyzed individuals who had undergone muscle biopsy (e.g., histopathology and/or electron transport chain activity results).

We performed protein 3D structure prediction for wild type and variant protein structures for variants of uncertain significance (VUS) in nDNA-PMD genes for predicting the impact of VUS on the protein structure. We retrieved protein FAST-All (FASTA) sequences from the UniProt database and input them to the AlphafoldColab2 program for 2D structure visualization and processing. We retrieved output data files from AlphafoldColab2 and input to PyMol 2.5.5 Edu for 3D structure visualization of the protein structures. We assessed sequence coverage and confidence levels for reliability and validity of structures. We performed all imaging of structures in accordance with the sequence alignment of variant protein structure with wild-type protein structures. We assessed variant protein models for predicted change in structure and amino-acid interaction in relation to wild type. We labelled variant residues for each gene variant.

We performed statistical analysis using R statistical software (version 4.0.2). Results are given as mean ± SD (range). Non-parametric Fisher’s exact test was chosen to compare between groups as indicated where appropriate. Results were considered statistically significant with a two-tailed p-value of < 0.05.

## Results

There were 403 individuals in our Excel database whom we identified from FoxPro, Sunquest, Connect Care and Metabolic Genetics Clinic Databases. Two-hundred-and ninety-seven individuals fulfilled the inclusion criteria. The demographics of all individuals are summarized in Table 1. All genetic and genomic investigations and their diagnostic yield was summarized in Table 2. All individuals with their phenotypes and genotypes are summarized in Supplemental Table 2 1 (mtDNA-PMD), Supplemental Table 2 (nDNA-PMD), and Supplemental Table 3 (non-PMD). All individuals with no genetic diagnosis are summarized in Supplemental Table 4.

**Table 1:** Demographic information of all individuals is summarized in Table 1

Demographics	Group 1	Group 2	Group 3	Group 4	Total
Numbers	Total (*n* = 41)	Total (*n* = 30)	Total (*n* = 22)	Total (*n* = 204)	Total (*n* = 297)
	Adult (*n* = 36)	Adult (*n* = 13)	Adult (*n* = 8)	Adult (*n* = 140)	Adult (*n* = 197)
	Children (*n* = 5)	Children (*n* = 17)	Children (*n* = 14)	Children (*n* = 64)	Children (*n* = 100)
Male	Total = 15	Total = 13	Total = 13	Total = 83	Total = 124
	Adult = 14	Adult = 7	Adult = 5	Adult = 48	Adult = 74
	Children = 1	Children = 6	Children = 8	Children = 35	Children = 50
Female	Total = 26	Total = 17	Total = 9	Total = 121	Total = 173
	Adult = 22	Adult = 6	Adult = 3	Adult = 92	Adult = 123
	Children = 4	Children = 11	Children = 6	Children = 29	Children = 50
Current age (range)	Total = 45.1 ± 19.5 SD yrs (6 mo–77 yrs)	Total = 19.8 ± 16.3 SD yrs (4 mo–51 yrs)	Total = 19.1 ± 18 SD yrs (1–71 yrs)	Total = 33.1 ± 22.5 SD yrs (6 wks–84 yrs)	Total = 32.4 ± 22.4 SD yrs (6 wks–84 yrs)
	Adult = 50 ± 15.2 SD yrs (21–77 yrs)	Adult = 34.8 ± 12.7 SD yrs (19–51 yrs)	Adult = 38.5 ± 15.9 SD yrs (19–71 yrs)	Adult = 44.2 ± 18.2 SD yrs (19–84 yrs)	Adult = 44.4 ± 17.6 SD yrs (19–84 yrs)
	Children = 9.9 ± 6.2 SD yrs (6 mo–15 yrs)	Children = 8.4 ± 6.6 SD yrs (4 mo–yrs)	Children = 8.1 ± 5.02 SD yrs (1.5–15 yrs)	Children = 8.8 ± 5.5 SD years (6 wks–18 yrs)	Children = 8.7 ± 5.6 SD years (6 wks–18 yrs)
Age of onset (range)	Total = 38.4 ± 17.8 SD yrs (3 mo–69 yrs)	Total = 9.5 ± 15.4 SD yrs (NB–58 yrs)	Total = 4.4 ± 7.8 SD yrs (NB–33 yrs)	Total = 20.9 ± 20.8 SD yrs (0–74 yrs)	Total = 20 ± 20.7 SD yrs (NB–74 yrs)
	Adult = 38.4 ± 17.8 SD yrs (18–69 yrs)	Adult = 20.9 ± 19.6 SD yrs (6 mo–49 yrs)	Adult = 19.6 ± 11 SD yrs (NB–33 yrs)	Adult = 31.4 ± 19.6 SD years (NB–74 years)	Adult = 31.1 ± 19.4 SD yrs (NB–74 yrs)
	Children = 1.3 ± 1.8 SD yrs (NB–4 yrs)	Children = 2.4 ± 3.8 SD yrs (NB–14 yrs)	Children = 1.9 ± 3.3 SD yrs (NB–10 yrs)	Children = 3.0 ± 3.7 SD yrs (NB–14 yrs)	Children = 2.6 ± 3.6 SD years (NB–14yrs)
Age of diagnosis (range)	Total = 38.8 ± 18.7 SD yrs (5 mo–75 yrs)	Total = 12.8 ± 15.3 SD yrs (NB–59 yrs)	Total = 13.6 ± 16.5 SD yrs (2 mo–65 yrs)	NA	Total = 24.7 ± 21.1 SD yrs (NB–75 yrs)
	Adult = 43.7 ± 13.9 SD yrs (19–75 yrs)	Adult = 24 ± 17.8 SD yrs (2 mo–59 yrs)	Adult = 28 ± 18.9 SD yrs (2–65 yrs)		Adult = 36.4 ± 17.9 SD yrs (2 mo–75 yrs)
	Children = 4.1 ± 4.4 SD yrs (NB–11 yrs)	Children = 4.7 ± 4.7 SD yrs (NB–12 yrs)	Children = 4.7 ± 3.9 SD yrs (2 mo–11 yrs)		Children = 4.5 ± 4.06 SD yrs (NB–12 yrs)
*Deceased*	Total = 5	Total = 3	Total = 5	Total = 26	Total = 39
	Adult = 4	Adult = 1	Adult = 1	Adult = 10	Adult = 16
	Children = 1	Children = 2	Children = 4	Children = 16	Children = 23
*Most common phenotypes*	Total = Muscular (*n* = 18)	Total = Muscular (*n* = 17)	Total = Neurodevelopmental (*n* = 17)	Total = Muscular (*n* = 102)	Total = Muscular (*n* = 150)
	Adult = Muscular (*n* = 16)	Adult = Muscular (*n* = 9)	Adult = Muscular (*n* = 5)	Adult = Muscular (n = 77)	Adult = Muscular (*n* = 104)
	Children = Neurodevelopmental (*n* = 4)	Children = Neurodevelopmental (*n* = 11)	Children = Neurodevelopmental (*n* = 13)	Children = Neurodevelopmental (*n* = 34)	Children = Neurodevelopmental (*n* = 62)

Mo(s) = Month(s); NB = Newborn; NA = Not applicable; Wks = Weeks; Yr(s) = Year(s)

**Table 2:** Diagnostic yield of molecular genetics and genomic investigations of all individuals are summarized in Table 2

	Diagnostic yield of clinical exome sequencing *n* (%)	Diagnostic yield of next generation sequencing panels *n* (%)	Diagnostic yield of mitochondrial genome sequencing *n* (%)	Diagnostic yield of mitochondrial common variant testing *n* (%)
Total	33 (37.1%)	9 (18.4%)	32 (15.8%)	8 (11.8%)
			Muscle mtDNA 17 (17.9%)	
			Urine mtDNA 14 (16.1%)	
			Blood mtDNA 4 (14.3%)	
			BuccalmtDNA 2 (50%)	
Adult	13 (25.5%)	3 (10.3%)	28 (20.7%)	6 (12.7%)
			Muscle mtDNA 15 (23.8%)	
			Urine mtDNA = 13 (22.4%)	
			Blood mtDNA 1 (5.9%)	
			Buccal mtDNA 1 (50%)	
Children	20 (52.6%)	6 (30%)	4 (6%)	2 (9.5%)
			Muscle mtDNA 2 (6.3%)	
			Urine mtDNA 1 (3.4%)	
			Blood mtDNA 1 (9%)	
			Buccal mtDNA 1 (50%)	

**Fig. 1:**
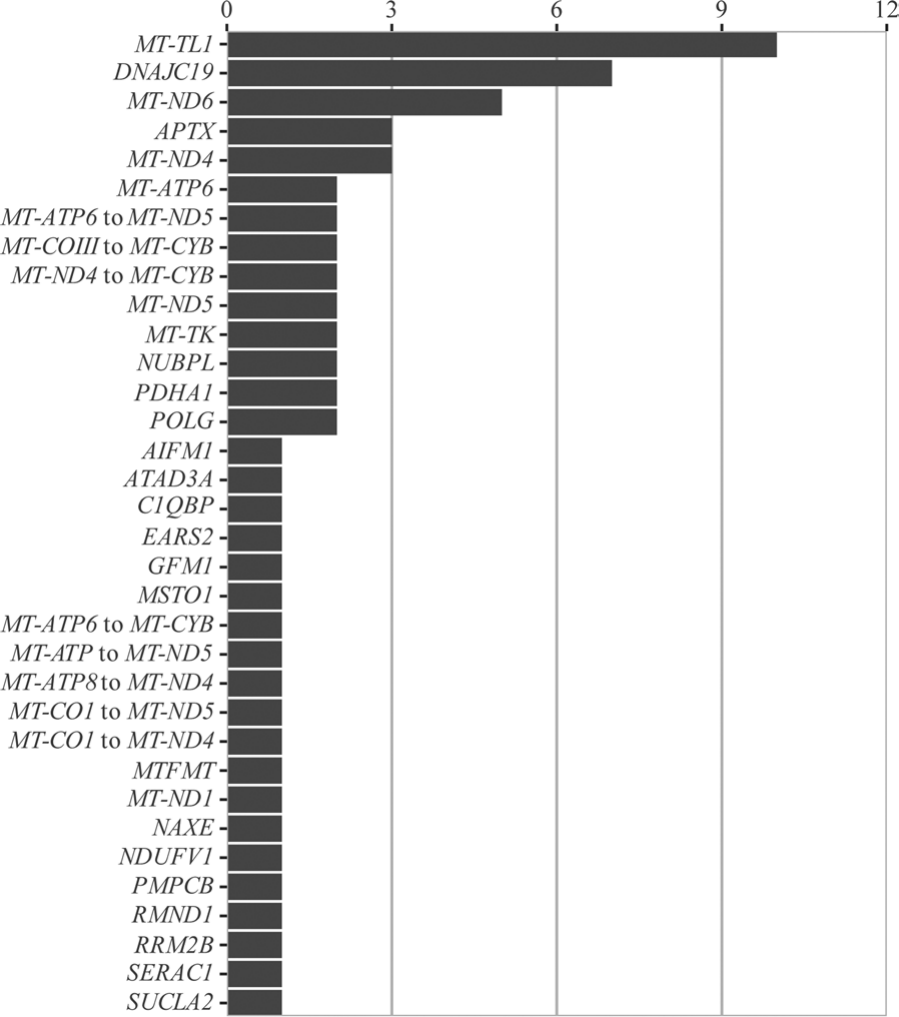
Genetic landscape of mtDNA-PMD and nDNA-PMD is depicted in Fig. 1

Ninety-three individuals had confirmed genetic diseases. The diagnostic yield of molecular genetics and genomic investigations was 31.3%. There were 71 individuals with PMD (mtDNA-PMD *n* = 41, nDNA-PMD *n* = 30), and 22 individuals with non-PMD. The number of individuals with mtDNA-PMD and nDNA-PMD is depicted in Fig. 1. There were 89 pathogenic/likely pathogenic single nucleotide variants in 45 genes in 98 individuals including 59 variants in 37 nuclear genes and 30 variants in eight mitochondrial genes. Additionally, there was one deletion spanning 137 nuclear genes and 18 mitochondrial deletions spanning two to 10 genes. All nDNA and mtDNA variants in previously established disease genes and their ACMG variant classification are summarized in Supplemental Table 5. There were five individuals who had common pathogenic variant (*n* = 1), multiple mtDNA deletions (*n* = 2) and large mtDNA single deletions (*n* = 2) with a < 20% heteroplasmy rate in muscle. There were seven VUS in six genes in six individuals including *TRIP12* (OMIM# 604506), *NEFH* (OMIM#162230), *RRM2B* (OMIM#604712), *NLRP3* (OMIM#606416), *POLG* (OMIM#174763), and *RYR1* (OMIM# 180901). These include two genes causing nDNA-PMD (*POLG*, *RRM2B*) and four genes causing non-PMD (*TRIP12*, *NEFH*, *NLRP3*, *RYR1*). Despite that the phenotypes of these individuals matched with their genotypes, due to the VUS classification of these variants, definitive genetic diagnoses were not confirmed (Supplemental Table 5). Additionally, we identified *PLCH2* (OMIM#612836) candidate gene in one individual (Supplemental Table 5). None of these individuals were included into the diagnostic yield calculations or Supplemental Tables 1, 2 and 3.

One hundred-three individuals underwent muscle biopsy (children *n* = 24 and adults *n* = 79). PMD was confirmed in 25 of those individuals (mitochondrial *n* = 16, nuclear *n* = 9). Five of those individuals had non-PMD. We depicted the number of molecular genetics and genomic investigations of these individuals in Supplemental Fig. 1.

### Phenotypes, biochemical features, and genotypes of individuals in group 1

This group includes 41 individuals with mtDNA-PMD (children *n* = 5; adults *n *= 36) from 40 families (Supplemental Table 5). We summarized their diagnoses, clinical features, biochemical features, neuroimaging, and genotypes in Supplemental Table 1. The number of individuals with different genetic diseases is depicted in Fig. 1. We included five individuals (Mito058; Mito214; Mito268; Mito282; Mito397) (Supplemental Table 1) with low heteroplasmy rate (< 20%) in different tissues (muscle *n* = 1; urine *n* = 2; blood *n* = 2). Only one of those individuals with a single large mtDNA deletion and a low heteroplasmy in muscle (Mito282) had a negative clinical exome sequencing. Despite low heteroplasmy rates, they were included into the Supplemental Table 1 and the diagnostic yield calculations as there are several reports in the medical literature that we summarized in the discussion.

Chronic progressive external ophthalmoplegia (CPEO) (OMIM#530000) was the most common phenotype (*n* = 17) in 41.5% of individuals with large mitochondrial DNA deletions. Muscle histopathology results revealed ragged red fibres (*n* = 10), reduced cytochrome c oxidase (COX) staining (*n* = 7), and mitochondrial abnormalities in electron microscopy (*n* = 10). 78% of individuals had a confirmed mtDNA-PMD by mitochondrial genome sequencing with or without deletion/duplication analysis and 87.5% of these genetic diagnoses were confirmed in muscle (*n* = 15) or urine (*n* = 14) mtDNA samples.

### Phenotypes, biochemical features, and genotypes of individuals in group 2

This group includes 30 individuals with nDNA-PMD (children *n* = 17; adults *n* = 13) from 25 families with 35 pathogenic/likely pathogenic variants in 19 different genes (Supplemental Table 5). We summarized their diagnoses, clinical features, biochemical features, neuroimaging, and genotypes in Supplemental Table 2. The number of individuals with different genetic diseases is depicted in Fig. 1.

The most common nDNA-PMD was dilated cardiomyopathy with ataxia syndrome (DCMA), also known as 3-methylglutaconic aciduria type V (OMIM#610198), due to biallelic pathogenic variants in *DNAJC19* (MIM#608977). There were seven affected individuals from four families. All individuals had elevated urine 3-methylglutaconic acid (if measured), and dilated cardiomyopathy or left ventricular dysfunction in echocardiography. Only one-third of individuals had ataxia. Cerebellar atrophy was reported in two out of four individuals who underwent brain MRI. One out of three individuals had hepatic steatosis in liver ultrasound.

Muscle histopathology results revealed ragged red fibres (n = 2), reduced COX staining (n = 3) and mitochondrial abnormalities in electron microscopy (n = 2). 63.3% of individuals had confirmed genetic diagnoses by clinical exome sequencing.

### Phenotypes, biochemical features and genotypes of individuals in group 3

This group includes 22 individuals with non-PMD from 21 families with 22 pathogenic/likely pathogenic variants. We summarized their diagnoses, clinical features, biochemical features, neuroimaging, and genotypes of individuals with non-PMD in Supplemental Table 3.

Muscle histopathology results revealed ragged red fibres (*n* = 1), reduced COX staining (*n* = 3), and mitochondrial abnormalities in electron microscopy (*n* = 2). 63.6% of individuals had confirmed genetic diagnoses by clinical exome sequencing.

### Phenotypes, biochemical features and genotypes of individuals in group 4

There were 204 individuals with suspected PMD without a genetic diagnosis. We summarized their phenotypes, biochemical investigations and molecular genetics and genomic investigations in Supplemental Table 4 and Supplemental Fig. 2. These individuals underwent extensive molecular genomic investigations including mitochondrial genome sequencing (72%), clinical exome sequencing (25.5%) and next generation sequencing panels (15.7%). It is still likely that some of these individuals may have a mtDNA- or nDNA-PMD.

### 3D protein structure prediction

The *RRM2B*, and *POLG* VUS showed shifts in their protein structures compared to the wildtype, with movements of large peripheral alpha helices measuring 17.5 and 7.8 angstroms, respectively. The structural analysis of these VUS are depicted in Supplemental Figs. 3 and 4.

### Comparison of individuals between groups

**Fig. 2:**
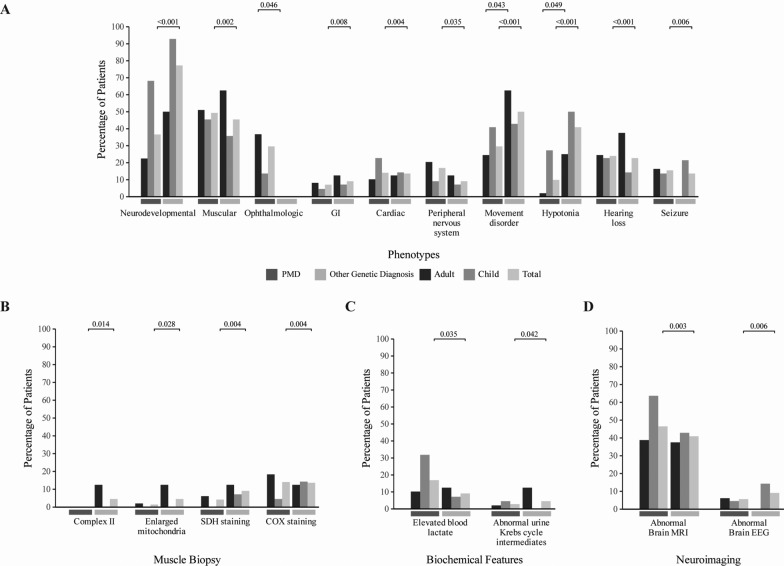
Comparison of phenotypes, biochemical features, muscle histopathology, electron transport chain activities, neuroimaging, and neurophysiological studies between all groups is depicted in Fig. 2

**Fig. 3:**
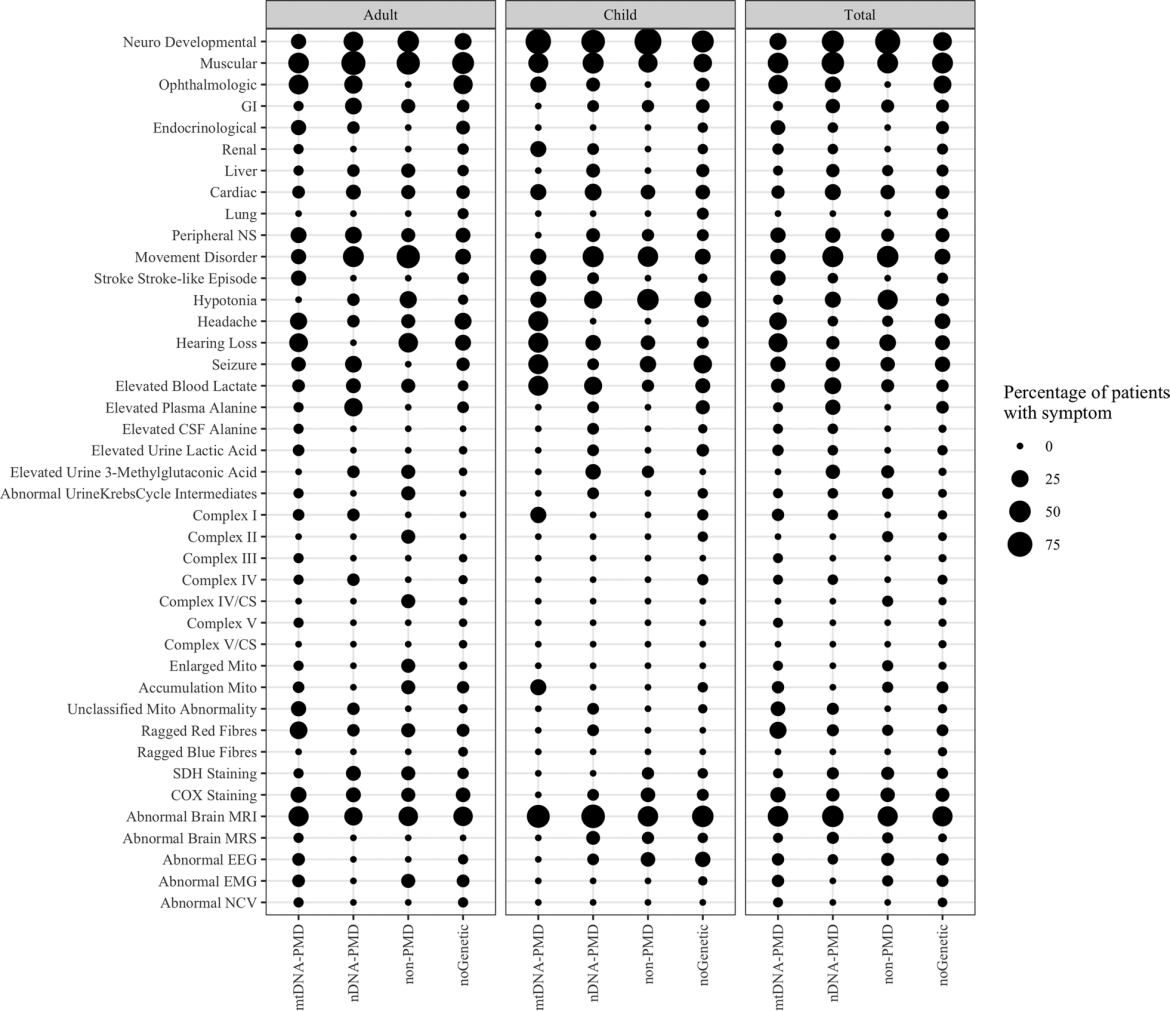
Comparison of clinical, biochemical features, muscle biopsy, and neuroimaging results between PMD (Groups 1 + 2) and non-PMD group (Group 3) is depicted in Fig. 3

We compared Groups 1, 2, and 3 for their phenotypes, biochemical features, muscle histopathology, electron transport chain activities, neuroimaging, and neurophysiological studies. We summarized all these information and their statistical analysis in Supplemental Table 6 and Supplemental Table 7. We depicted all these information in Fig. 2 and Supplemental Fig. 5. We combined Groups 1 and 2 (all with PMD) and compared with Group 3 (Supplemental Table 7 and Fig. 3). There was a statistically significant difference for muscular, cardiac, and ophthalmologic phenotypes, seizures, hearing loss, peripheral neuropathy in the Groups 1 + 2 compared to Group 3. Whereas there was a statistically significant difference for neurodevelopmental and gastrointestinal phenotypes, movement disorder and hypotonia in Group 3 compared to Group 1 + 2. The comparisons are depicted in Fig. 3 and Supplemental Table 7.

## Discussion

We report 71 individuals with 26 different mitochondrial and nuclear single gene PMD and 14 different mitochondrial genome deletions in our study cohort. Adults had higher percentage (87.8%) of mtDNA-PMD compared to children (*p*-value = 0.00123) in our study, which has been previously reported that about 30% of mtDNA-PMD are children [27–31]. Muscle histochemistry was suggestive of PMD in 44% of individuals with mtDNA-PMD, but only in 20% of individuals with nDNA-PMD. There is a statistically significant difference for neurodevelopmental phenotype between children and adults with PMD (*p* = 0.0004021). Although there is no statistically significant difference for muscular phenotype between children and adults, this phenotype is more common in adults. It has been previously reported that adults are more likely to present with classical PMD syndromes, whereas children present with non-specific [32] or neurodevelopmental phenotypes [32, 33]. We included four individuals with low heteroplasmy in either urine or in blood as they may have higher heteroplasmy in muscle. This was previously reported comparing muscle, urine and blood hetroplasmy rates that the muscle has the highest heteroplasmy rate [34–36]. We included two individuals: (1) one with a single large mtDNA deletion and a low heteroplasmy rate of 12% in muscle, who had normal clinical exome sequencing; (2) with multiple mtDNA deletions with a heteroplasmy rate of 20–30% as there were reports of low heteroplasmy in muscle for single or multiple mtDNA deletions [37–39]. We think that in the future more individuals with low heteroplasmy rate in muscle with single or multiple mtDNA deletions might be reported as mtDNA-PMD.

The diagnostic yield of clinical exome sequencing for suspected PMD has been reported between 35 and 57% in six different studies including children and/or adults [29, 40–45]. There were 28 to 142 individuals included into those studies. The number of confirmed genetic diseases ranged between 14 and 42. The diagnostic yield of targeted next generation sequencing panels for suspected PMD has been reported between 2.5 and 41% in five different studies including children and/or adults [46–52]. The number of genes on those next generation sequencing panels ranged between six and 1598. There were 42 to 450 individuals included in those studies. The number of confirmed genetic diseases ranged between six and 46 in those studies. The diagnostic yield of mitochondrial genome sequencing for suspected PMD has been reported between 2.4 and 35% in children and/or adults in four different studies [43, 47, 48, 52]. Two of those studies used blood samples and the diagnostic yields were between 7 and 12% [43, 48] and two of those studies used muscle samples and the diagnostic yields were between 2.4 and 35% [47, 52]. Detection of single mtDNA variants (m.3243A > G; m.8344A > G) [34, 36, 53] and single large-scale mtDNA deletions [34, 35] in urine were previously reported for the diagnosis of mtDNA-PMD. In a recent study, urine was sensitive to identify pathogenic mtDNA variants in nine out of 11 individuals with suspected PMD [54]. In our study, the diagnostic yield of clinical exome sequencing was 37.1%; the diagnostic yield of targeted next-generation sequencing panels was 18.4% and the diagnostic yield of mitochondrial genome sequencing was 15.8% including 17.9% in muscle mtDNA and 16.1% in urine mtDNA. To the best of our knowledge, we report for the first-time the diagnostic yield of urine mitochondrial genome sequencing in individuals with suspected PMD (*n* = 87) in the diagnosis of mtDNA-PMD. We think that the availability of urine mitochondrial genome sequencing in molecular genetics and genomic laboratories may be used as a first line cost effective and non-invasive screening test in adult individuals with suspected PMD.

Despite a similar number of children and adults underwent mitochondrial genome sequencing and nuclear genetics and genomic investigations, there were 36 adults with mtDNA-PMD whereas 17 children with nDNA-PMD (*p*-value = 0.00123). This is likely due to mitochondrial clonal expansion where pathogenic mitochondrial DNA variants (missense and deletions/duplications) replicate more than the wildtype mitochondrial DNA within a cell [55–57]. These pathogenic mitochondrial DNA variants accumulate over time and lead to a higher proportion of mitochondria carrying the same pathogenic variant within the cell and impair mitochondrial function [55–57]. There are several hypotheses for the mitochondrial clonal expansion. The random genetic drift hypothesis refers to pathogenic mitochondrial DNA variants accumulate by chance without selective advantage [58, 59]. The "survival of the smallest" [60] and "survival of the sickest" [61] hypotheses propose that smaller or less functional mitochondrial DNA molecules have a replicative advantage, either due to quicker replication or evasion of mitophagy. The negative feedback loop hypothesis proposes that reductions in mitochondrial DNA-encoded proteins lead to compensatory increases in mitochondrial DNA replication [62]. The perinuclear niche hypothesis suggests localized cellular responses to mitochondrial dysfunction near cell nuclei drive mitochondrial DNA replication through retrograde stress signaling [63]. Finally, a decline in mitophagy due to aging is associated with the accumulation of damaged mitochondria, which may explain the clonal expansion of pathogenic mitochondrial DNA variants [64, 65]. mtDNA variants are more likely to be lost in rapidly dividing cells such as blood, intestinal epithelium, buccal mucosa, and urine [55, 66–69]. However, it has been reported in some individuals that large-scale mtDNA deletions clonally expand from birth in skeletal muscle fibres and neurons in post-mitotic cells [55, 56, 70]. In our study, 66.7% of variants in muscle samples were deletions in adults with mtDNA-PMD. Interestingly, a study investigated muscle fibers in one individual and found that the number of mitochondria was increased in muscle fibers who had high number of mitochondrial DNA deletions and marked electron transport chain deficiencies in those fibers compared to the muscle fibers with less mitochondrial DNA deletions [71]. Mitochondrial DNA heteroplasmy increases with age due to accumulation of pathogenic mitochondrial DNA variants [72–74]. This may also explain why children have less mtDNA-PMD diagnosis in our study as they may have heteroplasmy rates lower than the detection limit of 10%.

DCMA is one of the rare nDNA-PMD due to biallelic pathogenic variants in *DNAJC19*. Less than 100 individuals with DCMA have been reported in the medical literature to date since its first description in 2006. In 2021, the c.130-1G > C pathogenic *DNAJC19* variant was reported in 43 individuals with DCMA from a Hutterite population in Alberta, Canada [75, 76]. The detailed phenotypes, neuroimaging, and long-term outcome information was available for about half of those individuals. Only one individual had macrovesicular steatosis, and moderate fibrosis in liver biopsy [75]. In a recent study, one individual with DCMA had fatty liver changes in liver ultrasound and severe steatosis and fibrosis in liver biopsy [77]. To the best of our knowledge, we report the third individual with DCMA who has hepatic steatosis in the liver ultrasound and five new individuals with DCMA for the first time in the medical literature. Interestingly, DNAJC19 plays a role in cardiolipin remodeling by binding with the prohibitin PHB2 molecule and modifying cardiolipin acylation resulting in the impairment of the integrity of the inner mitochondrial membrane [78–80]. Cardiolipin dysfunction impairs oxidative phosphorylation and increases the production of the reactive oxygen species (ROS) [80]. Additionally, hepatic steatosis is reported to be secondary to accumulation of fatty acids in liver cells and the exacerbation of oxidative stress and insulin resistance in PMD [81–83]. Mitochondrial ROS formation causes non-alcoholic steatosis in PMD [84]. There have been reports of hepatic disease (e.g. steatosis, fibrosis, cirrhosis) in about 20% of individuals with more than 70 different PMD [85, 86]. In our study, 5.6% of individuals with nDNA-PMD and mtDNA-PMD had hepatic disease (hepatic steatosis *n* = 1, hepatic cirrhosis *n* = 1, unspecified hepatic disease *n* = 2). It seems that there is a spectrum of liver phenotypes in PMD and ongoing oxidative stress, accumulation of fatty acids in liver and mitochondrial ROS formation result in a progressive hepatic disease leading to fibrosis and cirrhosis. For these reasons, we recommend close monitoring of liver in PMD for better understanding of the natural history of hepatic disease and management of disease morbidity.

There are other genetic diseases that mimic PMD [87]. Sometimes biochemical investigations cannot differentiate PMD from non-PMD [88]. Some of the non-PMD are spinal muscular atrophy (*SMN1*) [89], Friedreich ataxia (*FXN*) [90], Charcot-Marie-Tooth disease type 2 K (*GDAP1*) [89], hereditary spastic paraplegia 7 (*SPG7*), Wilson disease (*ATP7B*)[89], methylmalonic aciduria and propionic aciduria [91, 92], fatty acid oxidation disorders [93], argininosuccinic aciduria [94], purine and pyrimidine synthesis disorders [95], *BCAP31* associated encephalopathy [96] and riboflavin transporter deficiency (*SLC52A2*, * SLC52A3*) [97]. Some of these non-PMD are involved in mitochondrial energy metabolism and affect important co-factors for several enzymes such as Friedreich ataxia, riboflavin transporter deficiency, hereditary spastic paraplegia 7, and fatty acid oxidation disorders. Renal mitochondrial damage and altered mitochondrial energy metabolism was reported in propionic aciduria [98]. There is an overlap between PMD and non-PMD where the mitochondria are involved. Either accumulation or deficiency of organic molecules or cofactors affect the mitochondrial energy metabolism causing overlapping phenotypes (e.g., neurodevelopmental disorders, epilepsy, movement disorders) and biochemical features (e.g., elevated lactate, electron transport enzyme deficiencies) [87, 99–102]. There is an ongoing international collaboration for classification of metabolic genetic diseases [103, 104] that the list of PMD may be larger in the future. In our study cohort, 29% of individuals with confirmed genetic diseases had features suggestive of PMD (five with abnormal biochemical features suggestive of PMD). We summarized all causative genes causing and their protein–protein interactions with mitochondrial genes in our study cohort Supplemental Table 8 [44, 105–113]. There were 14 genes interacting with 32 mitochondrial genes associated with the electron transport chain, mitochondrial transcription regulation, mitochondrial ribosomal proteins, mitochondrial membrane transport and mitochondrial homeostasis (depicted in Supplemental Fig. 6). It is important to remember that the confirmation of molecular genetic diagnosis in PMD is crucial for the implication of the prognosis and management decisions. A misdiagnosis of PMD may prematurely end the diagnostic odyssey, overmedicalize the care and potentially limit access to appropriate treatments for the actual underlying genetic diseases. It is important to know that other genetic diseases can mimic PMD.

Our study had several limitations including: 1) It is a retrospective cohort study; 2) There were no detailed molecular genetics and genomic investigations for different phenotypes in several individuals; 3) We did not have biochemical investigations, muscle histopathology and electron transport chain enzyme activity measurements in the majority of our study cohort; 4) Adult muscle biopsy is part of clinical care at our center, which may explain why there are fewer children with muscle biopsy compared to adults. Despite these limitations, we report a large cohort of individuals with PMD and provide diagnostic yield of different molecular genetics and genomic investigations for the genetic diagnosis of PMD.

In conclusion we report a 23.7% diagnostic yield of molecular genetics and genomic investigations for the diagnosis of PMD in our cohort. We report the diagnostic yield of urine (16.1%) mitochondrial genome sequencing for the first time. We also report 71 individuals with 26 different mtDNA and nDNA single gene PMD and 14 different mtDNA deletions. Interestingly mtDNA-PMD was significantly more common in adults. We showed that liver phenotype might be progressive leading to cirrhosis which needs to be studied further by close monitoring of liver in PMD. We were able to show a direct relationship between some of the non-PMD genes and their indirect effects on mitochondrial machinery.

## Supplementary Information

Additional file 1.

Additional file 2.

Additional file 3.

Additional file 4.

Additional file 5.

Additional file 6.

Additional file 7.

Additional file 8.

Additional file 9.

## Data Availability

All data generated or analysed during this study are included in this published article and its supplementary information files.

## References

[1] Muraresku CC, McCormick EM, Falk MJ. Mitochondrial disease: advances in clinical diagnosis, management, therapeutic development, and preventative strategies. Curr Genet Med Rep. 2018;6:62–72. 30393588 10.1007/s40142-018-0138-9 PMC6208355

[2] Parikh S, Goldstein A, Karaa A, Koenig MK, Anselm I, Brunel-Guitton C, et al. Patient care standards for primary mitochondrial disease: a consensus statement from the mitochondrial medicine society. Genet Med. 2017;19:10. 10.1038/gim.2017.107 PMC7804217 28749475

[3] Saraste M. Oxidative phosphorylation at the fin de siècle. Science. 1999;283:1488–93. 10066163 10.1126/science.283.5407.1488

[4] Schapira AHV. Mitochondrial diseases. Lancet. 2012;379:1825–34. 22482939 10.1016/S0140-6736(11)61305-6

[5] Videla LA, Marimán A, Ramos B, José Silva M, del Campo A. Standpoints in mitochondrial dysfunction: underlying mechanisms in search of therapeutic strategies. Mitochondrion. 2022;63:9–22. 34990812 10.1016/j.mito.2021.12.006

[6] Koopman WJH, Willems PHGM, Smeitink JAM. Monogenic mitochondrial disorders. N Engl J Med. 2012;366:1132–41. 22435372 10.1056/NEJMra1012478

[7] Wallace DC, Chalkia D. Mitochondrial DNA genetics and the heteroplasmy conundrum in evolution and disease. Cold Spring Harbor Persp Biol. 2013;5(11):a021220. 10.1101/cshperspect.a021220 PMC3809581 24186072

[8] Mukherjee S, Ghosh A. Molecular mechanism of mitochondrial respiratory chain assembly and its relation to mitochondrial diseases. Mitochondrion. 2020;53:1–20. 32304865 10.1016/j.mito.2020.04.002

[9] Goldstein A, Rahman S. Seeking impact: Global perspectives on outcome measure selection for translational and clinical research for primary mitochondrial disorders. J Inherit Metab Dis. 2021;44:343–57. 33016339 10.1002/jimd.12320

[10] Keshavan N, Rahman S. Natural history of mitochondrial disorders: a systematic review. Essays Biochem. 2018;62:423–42. 29980629 10.1042/EBC20170108

[11] Bernier FP, Boneh A, Dennett X, Chow CW, Cleary MA, Thorburn DR. Diagnostic criteria for respiratory chain disorders in adults and children. Neurology. 2002;59(9):1406–11. 12427892 10.1212/01.wnl.0000033795.17156.00

[12] Morava E, van den Heuvel LP, Hol F, De Vries MC, Hogeveen M, Rodenburg RJ, Smeitink JA. Mitochondrial disease criteria: diagnostic applications in children. Neurology. 2006;67(10):1823–6. 17130416 10.1212/01.wnl.0000244435.27645.54

[13] Parikh S, Karaa A, Goldstein A, Bertini ES, Chinnery PF, Christodoulou J, et al. Diagnosis of possible’ mitochondrial disease: An existential crisis. J Med Genet. 2019;56:123–30. 30683676 10.1136/jmedgenet-2018-105800

[14] Emmanuele V, Ganesh J, Vladutiu G, Haas R, Kerr D, Saneto RP, et al. Time to harmonize mitochondrial syndrome nomenclature and classification: a consensus from the North American mitochondrial disease consortium (NAMDC). Mol Genet Metab. 2022;136:125–31. 35606253 10.1016/j.ymgme.2022.05.001 PMC9341219

[15] Schon KR, Ratnaike T, van den Ameele J, Horvath R, Chinnery PF. Mitochondrial diseases: a diagnostic revolution. Trends Genet. 2020;36:702–17. 32674947 10.1016/j.tig.2020.06.009

[16] Yamamoto K, Sakaue S, Matsuda K, Murakami Y, Kamatani Y, Ozono K, Momozawa Y, Okada Y. Genetic and phenotypic landscape of the mitochondrial genome in the Japanese population. Commun Biol. 2020;3(1):104. 32139841 10.1038/s42003-020-0812-9 PMC7058612

[17] Wong LJC. Next generation molecular diagnosis of mitochondrial disorders. Mitochondrion. 2013;13:379–87. 23473862 10.1016/j.mito.2013.02.001

[18] Disha B, Mathew RP, Dalal AB, Mahato AK, Satyamoorthy K, Singh KK, et al. Mitochondria in biology and medicine–2023. Mitochondrion. 2024;76:101853. 38423268 10.1016/j.mito.2024.101853

[19] Chen R, Aldred MA, Xu W, Zein J, Bazeley P, Comhair SAA, et al. Comparison of whole genome sequencing and targeted sequencing for mitochondrial DNA. Mitochondrion. 2021;58:303–10. 33513442 10.1016/j.mito.2021.01.006 PMC8354572

[20] Tranah GJ, Katzman SM, Lauterjung K, Yaffe K, Manini TM, Kritchevsky S, et al. Mitochondrial DNA m.3243A>G heteroplasmy affects multiple aging phenotypes and risk of mortality. Sci Rep. 2018;8:11887. 30089816 10.1038/s41598-018-30255-6 PMC6082898

[21] Bourgeois JM, Tarnopolsky MA. Pathology of skeletal muscle in mitochondrial disorders. Mitochondrion. 2004;4:441–52. 16120405 10.1016/j.mito.2004.07.036

[22] Gayathri N, Deepha S, Sharma S. Diagnosis of primary mitochondrial disorders-Emphasis on myopathological aspects. Mitochondrion. 2021;61:69–84. 34592422 10.1016/j.mito.2021.09.007

[23] Wong LJC. Diagnostic challenges of mitochondrial DNA disorders. Mitochondrion. 2007;7:45–52. 17276740 10.1016/j.mito.2006.11.025

[24] Richards S, Aziz N, Bale S, Bick D, Das S, Gastier-Foster J, et al. Standards and guidelines for the interpretation of sequence variants: a joint consensus recommendation of the American college of medical genetics and genomics and the association for molecular pathology. Genet Med. 2015;17:405–24. 25741868 10.1038/gim.2015.30 PMC4544753

[25] McCormick EM, Lott MT, Dulik MC, Shen L, Attimonelli M, Vitale O, et al. Specifications of the ACMG/AMP standards and guidelines for mitochondrial DNA variant interpretation. Hum Mutat. 2020;41:2028–57. 32906214 10.1002/humu.24107 PMC7717623

[26] Chen S, Francioli LC, Goodrich JK, Collins RL, Kanai M, Wang Q, et al. A genomic mutational constraint map using variation in 76,156 human genomes. Nature. 2024;625:92–100. 38057664 10.1038/s41586-023-06045-0 PMC11629659

[27] Koenig MK. Presentation and diagnosis of mitochondrial disorders in children. Pediatr Neurol. 2008;38:305–13. 18410845 10.1016/j.pediatrneurol.2007.12.001 PMC3099432

[28] Lamont PJ, Surtees R, Woodward CE, Leonard JV, Wood NW, Harding AE. Clinical and laboratory findings in referrals for mitochondrial DNA analysis. Arch Dis Childhood. 1998;79:22–7. 9771247 10.1136/adc.79.1.22 PMC1717633

[29] Taylor RW, Pyle A, Griffin H, Blakely EL, Duff J, He L, et al. Use of whole-exome sequencing to determine the genetic basis of multiple mitochondrial respiratory chain complex deficiencies. JAMA. 2014;312:68–77. 25058219 10.1001/jama.2014.7184 PMC6558267

[30] Thorburn DR. Mitochondrial disorders: prevalence, myths and advances. J Inherit Metab Dis. 2004;27:349–62. 15190193 10.1023/B:BOLI.0000031098.41409.55

[31] DiMauro S, Hirano M. Mitochondrial encephalomyopathies: an update. Neuromuscul Disord. 2005;15:276–86. 15792866 10.1016/j.nmd.2004.12.008

[32] Rahman S. Mitochondrial disease in children. J Intern Med. 2020;287:609–33. 32176382 10.1111/joim.13054

[33] Munnich A, Rotig A, Chretien D, Cormier V, Bourgeron T, Bonnefont J-R, et al. Clinical presentation of mitochondrial disorders in childhood. J Inher Metab Dis. 1996;19:521–7. 8884575 10.1007/BF01799112

[34] Blackwood JK, Whittaker RG, Blakely EL, Alston CL, Turnbull DM, Taylor RW. The investigation and diagnosis of pathogenic mitochondrial DNA mutations in human urothelial cells. Biochem Biophys Res Commun. 2010;393:740–5. 20171163 10.1016/j.bbrc.2010.02.072

[35] Varhaug KN, Nido GS, de Coo I, Isohanni P, Suomalainen A, Tzoulis C, et al. Using urine to diagnose large-scale mtDNA deletions in adult patients. Ann Clin Transl Neurol. 2020;7:1318–26. 32634300 10.1002/acn3.51119 PMC7448145

[36] McDonnell MT, Schaefer AM, Blakely EL, McFarland R, Chinnery PF, Turnbull DM, et al. Nonivasive diagnosis of the 3243A>G mitochondrial DNA mutation using urinary epithelial cells. Eur J Hum Genet. 2004;12:778–81. 15199381 10.1038/sj.ejhg.5201216

[37] Carey AR, Miller NR, Cui H, Allis K, Balog A, Bai R, et al. Myopathy and ophthalmologic abnormalities in association with multiple skeletal muscle mitochondrial DNA deletions. J Neuroophthalmol. 2024;44:247–52. 37665646 10.1097/WNO.0000000000001984

[38] Grady JP, Campbell G, Ratnaike T, Blakely EL, Falkous G, Nesbitt V, et al. Disease progression in patients with single, large-scale mitochondrial DNA deletions. Brain. 2014;137:323–34. 24277717 10.1093/brain/awt321 PMC3914470

[39] Leung DG, Cohen JS, Michelle EH, Bai R, Mammen AL, Christopher-Stine L. Mitochondrial DNA deletions with low-level heteroplasmy in adult-onset myopathy. J Clin Neuromuscul Dis. 2018;19:117–23. 29465611 10.1097/CND.0000000000000200 PMC5824425

[40] Ohtake A, Murayama K, Mori M, Harashima H, Yamazaki T, Tamaru S, et al. Diagnosis and molecular basis of mitochondrial respiratory chain disorders: exome sequencing for disease gene identification. Biochim Biophys Acta Gen Subj. 2014;1840:1355–9. 10.1016/j.bbagen.2014.01.025 24462578

[41] Pronicka E, Piekutowska-Abramczuk D, Ciara E, Trubicka J, Rokicki D, Karkucińska-Więckowska A, Pajdowska M, Jurkiewicz E, Halat P, Kosińska J, Pollak A. New perspective in diagnostics of mitochondrial disorders: two years’ experience with whole-exome sequencing at a national paediatric centre. J Transl Med. 2016;14:1–9. 27290639 10.1186/s12967-016-0930-9 PMC4903158

[42] Wortmann SB, Koolen DA, Smeitink JA, van den Heuvel L, Rodenburg RJ. Whole exome sequencing of suspected mitochondrial patients in clinical practice. J Inherit Metab Dis. 2015;38:437–43. 25735936 10.1007/s10545-015-9823-y PMC4432107

[43] Kohda M, Tokuzawa Y, Kishita Y, Nyuzuki H, Moriyama Y, Mizuno Y, Hirata T, Yatsuka Y, Yamashita-Sugahara Y, Nakachi Y, Kato H. A comprehensive genomic analysis reveals the genetic landscape of mitochondrial respiratory chain complex deficiencies. PLoS Genet. 2016;12(1):e1005679. 26741492 10.1371/journal.pgen.1005679 PMC4704781

[44] Puusepp S, Reinson K, Pajusalu S, Murumets Ü, Õiglane-Shlik E, Rein R, et al. Effectiveness of whole exome sequencing in unsolved patients with a clinical suspicion of a mitochondrial disorder in Estonia. Mol Genet Metab Rep. 2018;15:80–9. 30009132 10.1016/j.ymgmr.2018.03.004 PMC6043467

[45] Rogac M, Neubauer D, Leonardis L, Pecaric N, Meznaric M, Maver A, et al. Clinical experience of neurological mitochondrial diseases in children and adults: a single-center study. Balkan J Med Genet. 2021;24:5–14. 36249517 10.2478/bjmg-2021-0019 PMC9524181

[46] Dare JT, Vasta V, Penn J, Tran NTB, Hahn SH. Targeted exome sequencing for mitochondrial disorders reveals high genetic heterogeneity. BMC Med Genet. 2013;14:118. 24215330 10.1186/1471-2350-14-118 PMC3827825

[47] Ardissone A, Bruno C, Diodato D, Donati A, Ghezzi D, Lamantea E, et al. Clinical, imaging, biochemical and molecular features in Leigh syndrome: a study from the Italian network of mitochondrial diseases. Orphanet J Rare Dis. 2021;16:1. 34627336 10.1186/s13023-021-02029-3 PMC8501644

[48] Lieber DS, Calvo SE, Shanahan K, Slate NG, Liu S, Hershman SG, et al. Targeted exome sequencing of suspected mitochondrial disorders. Neurology. 2013;80(19):1762–70. 23596069 10.1212/WNL.0b013e3182918c40 PMC3719425

[49] McKiernan P, Ball S, Santra S, Foster K, Fratter C, Poulton J, et al. Incidence of primary mitochondrial disease in children younger than 2 years presenting with acute liver failure. J Pediatr Gastroenterol Nutr. 2016;63:592–7. 27482763 10.1097/MPG.0000000000001345 PMC5113754

[50] Nogueira C, Silva L, Pereira C, Vieira L, Leão Teles E, Rodrigues E, et al. Targeted next generation sequencing identifies novel pathogenic variants and provides molecular diagnoses in a cohort of pediatric and adult patients with unexplained mitochondrial dysfunction. Mitochondrion. 2019;47:309–17. 30831263 10.1016/j.mito.2019.02.006

[51] Nogueira C, Pereira C, Silva L, Laranjeira M, Lopes A, Neiva R, et al. The genetic landscape of mitochondrial diseases in the next-generation sequencing era: a Portuguese cohort study. Front Cell Dev Biol. 2024;12:1331351. 38465286 10.3389/fcell.2024.1331351 PMC10920333

[52] Calvo SE, Compton AG, Hershman SG, Lim SC, Lieber DS, Tucker EJ, et al. Molecular diagnosis of infantile mitochondrial disease with targeted next-generation sequencing. Sci Transl Med. 2012;4:118ra10. 22277967 10.1126/scitranslmed.3003310 PMC3523805

[53] Hammans SR, Sweeney MG, Hanna MG, Brockington M, Morgan-Hughes JA, Harding AE. The mitochondria! DNA transfer RNA Leu<UUR) a clinical and genetic study. Brain. 1995;118:721–34. 7600089 10.1093/brain/118.3.721

[54] Mavraki E, Labrum R, Sergeant K, Alston CL, Woodward C, Smith C, et al. Genetic testing for mitochondrial disease: the United Kingdom best practice guidelines. Eur J Hum Genet. 2023;31:148–63. 36513735 10.1038/s41431-022-01249-w PMC9905091

[55] Lawless C, Greaves L, Reeve AK, Turnbull DM, Vincent AE. The rise and rise of mitochondrial DNA mutations. Open Biol Royal Soc Publ. 2020;10(5):200061. 10.1098/rsob.200061 PMC7276526 32428418

[56] Campbell G, Krishnan KJ, Deschauer M, Taylor RW, Turnbull DM. Dissecting the mechanisms underlying the accumulation of mitochondrial DNA deletions in human skeletal muscle. Hum Mol Genet. 2014;23:4612–20. 24740879 10.1093/hmg/ddu176 PMC4119413

[57] Larsson N-G. Somatic mitochondrial DNA mutations in mammalian aging. Annu Rev Biochem. 2010;79:683–706. 20350166 10.1146/annurev-biochem-060408-093701

[58] Chinnery PF, Samuels DC. Relaxed replication of mtDNA: a model with implications for the expression of disease. Am J Hum Genet. 1999;64:1158–65. 10090901 10.1086/302311 PMC1377840

[59] Elson JL, Samuels DC, Turnbull DM, Chinnery PF. Random intracellular drift explains the clonal expansion of mitochondrial DNA mutations with age. Am J Hum Genet. 2001;68:802–6. 11179029 10.1086/318801 PMC1274494

[60] Wallace DC. Mitochondrial DNA mutations and neuromuscular disease. Trends Genet. 1989;5:9–13. 2652392 10.1016/0168-9525(89)90005-x

[61] Yoneda M, Chomyn A, Martinuzzi A, Hurkot O, Attardi G. Marked replicative advantage of human mtDNA carrying a point mutation that causes the MELAS encephalomyopathy. Proc Natl Acad Sci U S A. 1992;89(23):11164–8. 1454794 10.1073/pnas.89.23.11164 PMC50510

[62] Kowald A, Kirkwood TBL. Transcription could be the key to the selection advantage of mitochondrial deletion mutants in aging. Proc Natl Acad Sci U S A. 2014;111:2972–7. 24569805 10.1073/pnas.1314970111 PMC3939916

[63] Vincent AE, Rosa HS, Pabis K, Lawless C, Chen C, Grünewald A, et al. Subcellular origin of mitochondrial DNA deletions in human skeletal muscle. Ann Neurol. 2018;84:289–301. 30014514 10.1002/ana.25288 PMC6141001

[64] Cuervo AM, Bergamini E, Brunk UT, Dröge W, Ffrench M, Terman A. Autophagy and aging: the importance of maintaining “clean” cells. Autophagy. 2005;1:131–40. 16874025 10.4161/auto.1.3.2017

[65] Li H, Slone J, Huang T. The role of mitochondrial-related nuclear genes in age-related common disease. Mitochondrion. 2020;53:38–47. 32361035 10.1016/j.mito.2020.04.012

[66] Olsson C, Johnsen E, Nilsson M, Wilander E, Ènen A-CS, Lagerstro M, et al. The level of the mitochondrial mutation A3243G decreases upon ageing in epithelial cells from individuals with diabetes and deafness. Euro J Human Genet. 2001;9(12):917–21. 10.1038/sj.ejhg.5200742 11840193

[67] Grady JP, Pickett SJ, Ng YS, Alston CL, Blakely EL, Hardy SA, Feeney CL, Bright AA, Schaefer AM, Gorman GS, McNally RJ. mt DNA heteroplasmy level and copy number indicate disease burden in m. 3243A>G mitochondrial disease. EMBO Mol Med. 2018;10(6):e8262. 29735722 10.15252/emmm.201708262 PMC5991564

[68] Rahman S, Poulton J, Marchington D, Suomalainen A. Decrease of 3243 ArG mtDNA mutation from blood in MELAS syndrome: a longitudinal study. Am J Hum Genet. 2001;68:238–40. 11085913 10.1086/316930 PMC1234919

[69] Frederiksen AL, Andersen PH, Kyvik KO, Jeppesen TD, Vissing J, Schwartz M. Tissue specific distribution of the 3243A→G mtDNA mutation. J Med Genet. 2006;43:671–7. 16490799 10.1136/jmg.2005.039339 PMC2564591

[70] Brockington M, Alsanjari N, Sweeney MG, Morgan-Hughes JA, Scaravilli F, Harding AE. Kearns–Sayre syndrome associated with mitochondrial DNA deletion or duplication: a molecular genetic and pathological study. J Neurol Sci. 1995;131:78–87. 7561952 10.1016/0022-510x(95)00091-f

[71] Rocha MC, Rosa HS, Grady JP, Blakely EL, He L, Romain N, et al. Pathological mechanisms underlying single large-scale mitochondrial DNA deletions. Ann Neurol. 2018;83:115–30. 29283441 10.1002/ana.25127 PMC5893934

[72] Sondheimer N, Glatz CE, Tirone JE, Deardorff MA, Krieger AM, Hakonarson H. Neutral mitochondrial heteroplasmy and the influence of aging. Hum Mol Genet. 2011;20:1653–9. 21296868 10.1093/hmg/ddr043 PMC3063991

[73] Liu C, Fetterman JL, Qian Y, Sun X, Blackwell TW, Pitsillides A, et al. Presence and transmission of mitochondrial heteroplasmic mutations in human populations of European and African ancestry. Mitochondrion. 2021;60:33–42. 34303007 10.1016/j.mito.2021.07.004 PMC8464516

[74] Bornstein R, Gonzalez B, Johnson SC. Mitochondrial pathways in human health and aging. Mitochondrion. 2020;54:72–84. 32738358 10.1016/j.mito.2020.07.007 PMC7508824

[75] Machiraju P, Degtiarev V, Patel D, Hazari H, Lowry RB, Bedard T, et al. Phenotype and pathology of the dilated cardiomyopathy with ataxia syndrome in children. J Inherit Metab Dis. 2022;45:366–76. 34580891 10.1002/jimd.12441

[76] Chong JX, Ouwenga R, Anderson RL, Waggoner DJ, Ober C. A population-based study of autosomal-recessive disease-causing mutations in a founder population. Am J Hum Genet. 2012;91:608–20. 22981120 10.1016/j.ajhg.2012.08.007 PMC3484657

[77] Papadopoulou-Legbelou K, Ntoumpara M, Kavga M, Kotanidou EP, Papoulidis I, Galli-Tsinopoulou A, et al. Genital abnormalities and growth retardation as early signs of dilated cardiomyopathy with ataxia syndrome. Case Rep Genet. 2024;2024:1–7. 10.1155/2024/8860889 PMC10821800 38283849

[78] Richter-Dennerlein R, Korwitz A, Haag M, Tatsuta T, Dargazanli S, Baker M, et al. DNAJC19, a mitochondrial cochaperone associated with cardiomyopathy, forms a complex with prohibitins to regulate cardiolipin remodeling. Cell Metab. 2014;20:158–71. 24856930 10.1016/j.cmet.2014.04.016

[79] Mileykovskaya E, Zhang M, Dowhan W. Cardiolipin in energy transducing membranes. Biochemistry (Mosc). 2005;70:154–8. 15807653 10.1007/s10541-005-0095-2

[80] Paradies G, Paradies V, Ruggiero FM, Petrosillo G. Oxidative stress, cardiolipin and mitochondrial dysfunction in nonalcoholic fatty liver disease. World J Gastroenterol WJG Press. 2014;20:14205–18. 10.3748/wjg.v20.i39.14205 PMC4202349 25339807

[81] Nassir F, Ibdah JA. Role of mitochondria in nonalcoholic fatty liver disease. Int J Mol Sci MDPI. 2014;15(5):8713–42. 10.3390/ijms15058713 PMC4057755 24837835

[82] Nassir F, Scott Rector R, Hammoud GM, Ibdah JA. Pathogenesis and prevention of hepatic steatosis. Gastroenterol Hepatol (N Y). 2015;11(3):167–75. 27099587 PMC4836586

[83] Rector RS, Thyfault JP, Uptergrove GM, Morris EM, Naples SP, Borengasser SJ, et al. Mitochondrial dysfunction precedes insulin resistance and hepatic steatosis and contributes to the natural history of non-alcoholic fatty liver disease in an obese rodent model. J Hepatol. 2010;52:727–36. 20347174 10.1016/j.jhep.2009.11.030 PMC3070177

[84] Pérez-Carreras M, Del Hoyo P, Martín MA, Rubio JC, Martín A, Castellano G, et al. Defective hepatic mitochondrial respiratory chain in patients with nonalcoholic steatohepatitis. Hepatology. 2003;38:999–1007. 14512887 10.1053/jhep.2003.50398

[85] Naess K (2017) Mitochondrial disease in children-from clinical presentation to genetic background

[86] Ayers M, Horslen SP, Gómez AM, Squires JE. Mitochondrial Hepatopathy. Clin Liver Dis. 2022;26(3):421–38. 35868683 10.1016/j.cld.2022.03.006

[87] Niyazov DM, Kahler SG, Frye RE. Primary mitochondrial disease and secondary mitochondrial dysfunction: importance of distinction for diagnosis and treatment. Mol Syndromol S Karger AG. 2016;7:122–37. 10.1159/000446586 PMC4988248 27587988

[88] Parikh S, Goldstein A, Koenig MK, Scaglia F, Enns GM, Saneto R, et al. Diagnosis and management of mitochondrial disease: a consensus statement from the mitochondrial medicine society. Genet Med. 2015;17:689–701. 25503498 10.1038/gim.2014.177 PMC5000852

[89] Katsetos CD, Koutzaki S, Melvin JJ. Mitochondrial dysfunction in neuromuscular disorders. Semin Pediatr Neurol. 2013;20:202–15. 24331362 10.1016/j.spen.2013.10.010

[90] Calabrese V, Lodi R, Tonon C, D’Agata V, Sapienza M, Scapagnini G, et al. Oxidative stress, mitochondrial dysfunction and cellular stress response in Friedreich’s ataxia. J Neurol Sci. 2005;233:145–62. 15896810 10.1016/j.jns.2005.03.012

[91] de Keyzer Y, Valayannopoulos V, Benoist J-F, Batteux F, Lacaille F, Hubert L, et al. Multiple OXPHOS deficiency in the liver, kidney, heart, and skeletal muscle of patients with methylmalonic aciduria and propionic aciduria. Pediatr Res. 2009;66:91–5. 19342984 10.1203/PDR.0b013e3181a7c270

[92] Baruteau J, Hargreaves I, Krywawych S, Chalasani A, Land JM, Davison JE, et al. Successful reversal of propionic acidaemia associated cardiomyopathy: evidence for low myocardial coenzyme Q10 status and secondary mitochondrial dysfunction as an underlying pathophysiological mechanism. Mitochondrion. 2014;17:150–6. 25010387 10.1016/j.mito.2014.07.001

[93] Nsiah-Sefaa A, McKenzie M. Combined defects in oxidative phosphorylation and fatty acid β-oxidation in mitochondrial disease. Biosci Rep. 2016;36:e00313. 26839416 10.1042/BSR20150295 PMC4793296

[94] Monné M, Miniero DV, Daddabbo L, Palmieri L, Porcelli V, Palmieri F. Mitochondrial transporters for ornithine and related amino acids: a review. Amino Acids. 2015;47:1763–77. 26002808 10.1007/s00726-015-1990-5

[95] Duley JA, Christodoulou J, de Brouwer APM. The PRPP synthetase spectrum: what does it demonstrate about nucleotide syndromes? Nucleosides Nucleotides Nucleic Acids. 2011;30:1129–39. 22132967 10.1080/15257770.2011.591747

[96] Albanyan S, Al Teneiji A, Monfared N, Mercimek-Mahmutoglu S. BCAP31-associated encephalopathy and complex movement disorder mimicking mitochondrial encephalopathy. Am J Med Genet A. 2017;173:1640–3. 28332767 10.1002/ajmg.a.38127

[97] Nimmo GAM, Ejaz R, Cordeiro D, Kannu P, Mercimek-Andrews S. Riboflavin transporter deficiency mimicking mitochondrial myopathy caused by complex II deficiency. Am J Med Genet A. 2018;176:399–403. 29193829 10.1002/ajmg.a.38530

[98] Schumann A, Belche V, Schaller K, Grünert SC, Kaech A, Baumgartner MR, et al. Mitochondrial damage in renal epithelial cells is potentiated by protein exposure in propionic aciduria. J Inherit Metab Dis. 2021;44:1330–42. 34297429 10.1002/jimd.12419

[99] Zolkipli Z, Sherlock M, Biggar WD, Taylor G, Hutchison JS, Peliowski A, et al. Abnormal fatty acid metabolism in spinal muscular atrophy may predispose to perioperative risks. Eur J Paediatr Neurol. 2012;16:549–53. 22264649 10.1016/j.ejpn.2012.01.004

[100] Berger A, Mayr JA, Meierhofer D, Fötschl U, Bittner R, Budka H, et al. Severe depletion of mitochondrial DNA in spinal muscular atrophy. Acta Neuropathol. 2003;105:245–51. 12557011 10.1007/s00401-002-0638-1

[101] Rossignol DA, Frye RE. A review of research trends in physiological abnormalities in autism spectrum disorders: immune dysregulation, inflammation, oxidative stress, mitochondrial dysfunction and environmental toxicant exposures. Mol Psychiatry. 2012;17:389–401. 22143005 10.1038/mp.2011.165 PMC3317062

[102] Slane BG, Aykin-Burns N, Smith BJ, Kalen AL, Goswami PC, Domann FE, et al. Mutation of succinate dehydrogenase subunit C results in increased O2, oxidative stress, and genomic instability. Cancer Res. 2006;66:7615–20. 16885361 10.1158/0008-5472.CAN-06-0833

[103] Ferreira CR, van Karnebeek CDM, Vockley J, Blau N. A proposed nosology of inborn errors of metabolism. Genet Med. 2019;21:102–6. 29884839 10.1038/s41436-018-0022-8 PMC6286709

[104] Ferreira CR, Rahman S, Keller M, Zschocke J, ICIMD Advisory Group (2021) An international classification of inherited metabolic disorders (ICIMD). J Inherit Metab Dis. 44:164–77 10.1002/jimd.12348 PMC9021760 33340416

[105] Balicza P, Gezsi A, Fedor M, Sagi JC, Gal A, Varga NA, et al. Multilevel evidence of MECP2-associated mitochondrial dysfunction and its therapeutic implications. Front Psychiatry. 2023;14:1301272. 38250256 10.3389/fpsyt.2023.1301272 PMC10796460

[106] Huttlin EL, Ting L, Bruckner RJ, Gebreab F, Gygi MP, Szpyt J, et al. The bioplex network: a systematic exploration of the human interactome. Cell. 2015;162:425–40. 26186194 10.1016/j.cell.2015.06.043 PMC4617211

[107] Huttlin EL, Bruckner RJ, Paulo JA, Cannon JR, Ting L, Baltier K, et al. Architecture of the human interactome defines protein communities and disease networks. Nature. 2017;545:505–9. 28514442 10.1038/nature22366 PMC5531611

[108] Havugimana PC, Hart GT, Nepusz T, Yang H, Turinsky AL, Li Z, et al. A census of human soluble protein complexes. Cell. 2012;150:1068–81. 22939629 10.1016/j.cell.2012.08.011 PMC3477804

[109] Hein MY, Hubner NC, Poser I, Cox J, Nagaraj N, Toyoda Y, et al. A human interactome in three quantitative dimensions organized by stoichiometries and abundances. Cell. 2015;163:712–23. 26496610 10.1016/j.cell.2015.09.053

[110] Browning R, Karim S. RNA interference-mediated depletion of N-ethylmaleimide sensitive fusion protein and synaptosomal associated protein of 25 kDa results in the inhibition of blood feeding of the Gulf Coast tick *Amblyomma maculatum*. Insect Mol Biol. 2013;22:245–57. 23437815 10.1111/imb.12017 PMC3644323

[111] Kahle JJ, Gulbahce N, Shaw CA, Lim J, Hill DE, Barabási A-L, et al. Comparison of an expanded ataxia interactome with patient medical records reveals a relationship between macular degeneration and ataxia. Hum Mol Genet. 2011;20:510–27. 21078624 10.1093/hmg/ddq496 PMC3016911

[112] Garcia-Esparcia P, López-González I, Grau-Rivera O, García-Garrido MF, Konetti A, Llorens F, et al. Dementia with lewy bodies: molecular pathology in the frontal cortex in typical and rapidly progressive forms. Front Neurol. 2017;8:89. 28348546 10.3389/fneur.2017.00089 PMC5346561

[113] Jha V, Roy B, Jahagirdar D, McNutt ZA, Shatoff EA, Boleratz BL, et al. Structural basis of sequestration of the anti-Shine-Dalgarno sequence in the Bacteroidetes ribosome. Nucleic Acids Res. 2021;49:547–67. 33330920 10.1093/nar/gkaa1195 PMC7797042

