# Supplementary figures and images for: Genetic landscape of primary mitochondrial diseases in children and adults using molecular genetics and genomic investigations of mitochondrial and nuclear genome
Source: Orphanet J Rare Dis. 2024 Nov 12;19:424. doi: 10.1186/s13023-024-03437-x (PMC11555972; doi:10.1186/s13023-024-03437-x)

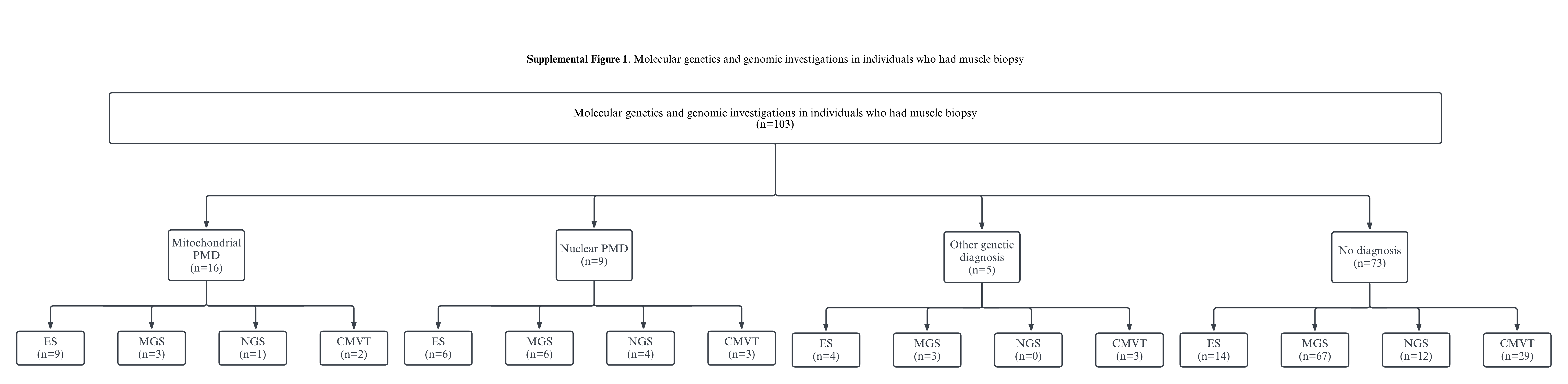

Supplement: Supplementary file 1 — Additional file 1. [file 13023_2024_3437_MOESM1_ESM.tiff]

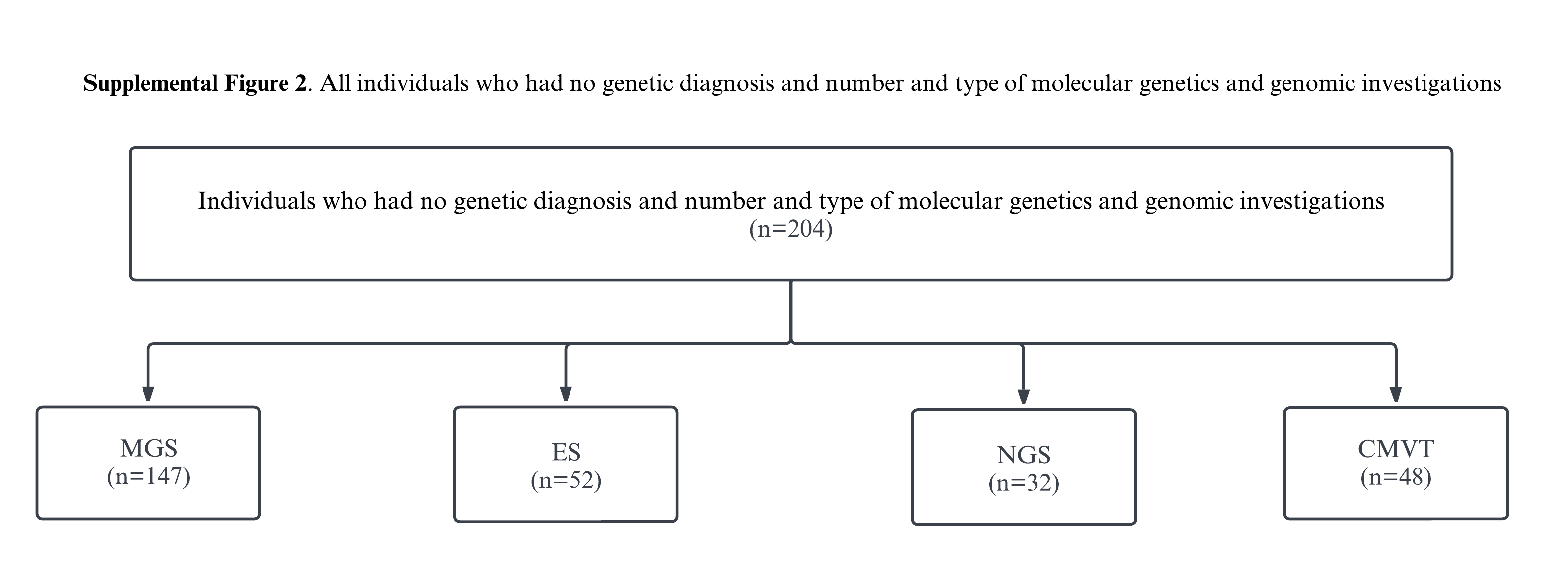

Supplement: Supplementary file 2 — Additional file 2. [file 13023_2024_3437_MOESM2_ESM.tiff]

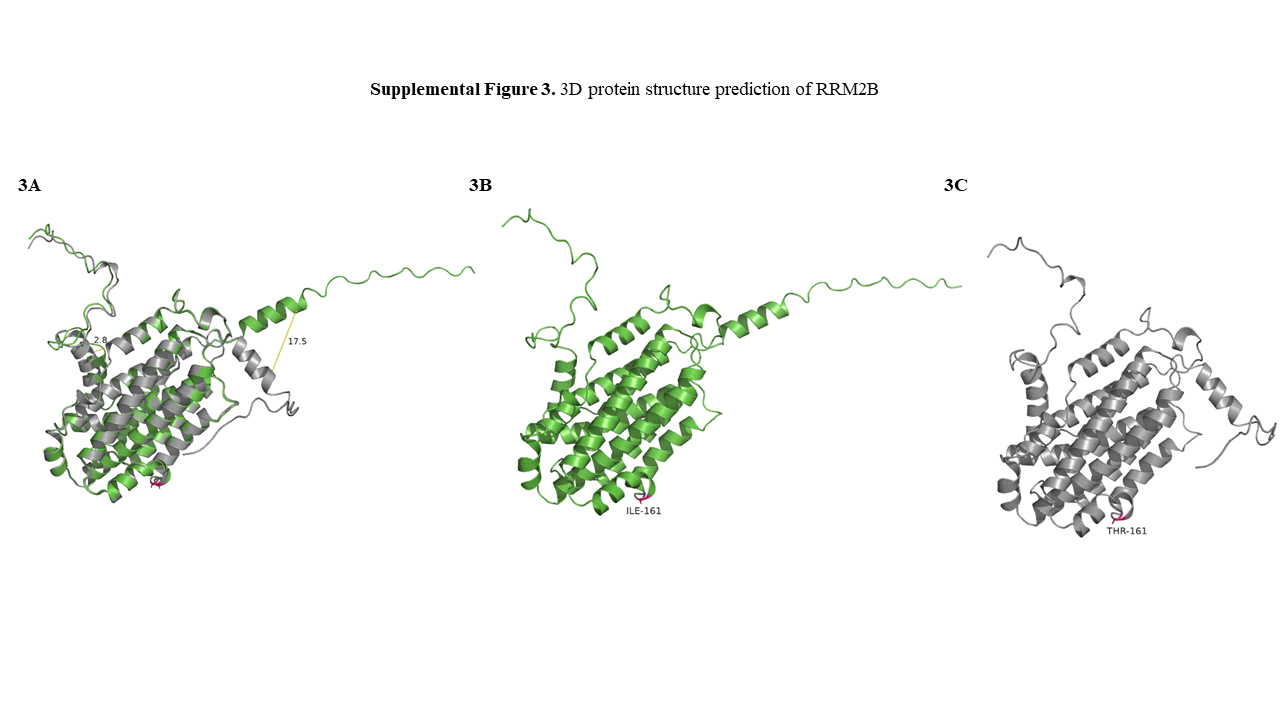

Supplement: Supplementary file 3 — Additional file 3. [file 13023_2024_3437_MOESM3_ESM.tiff]

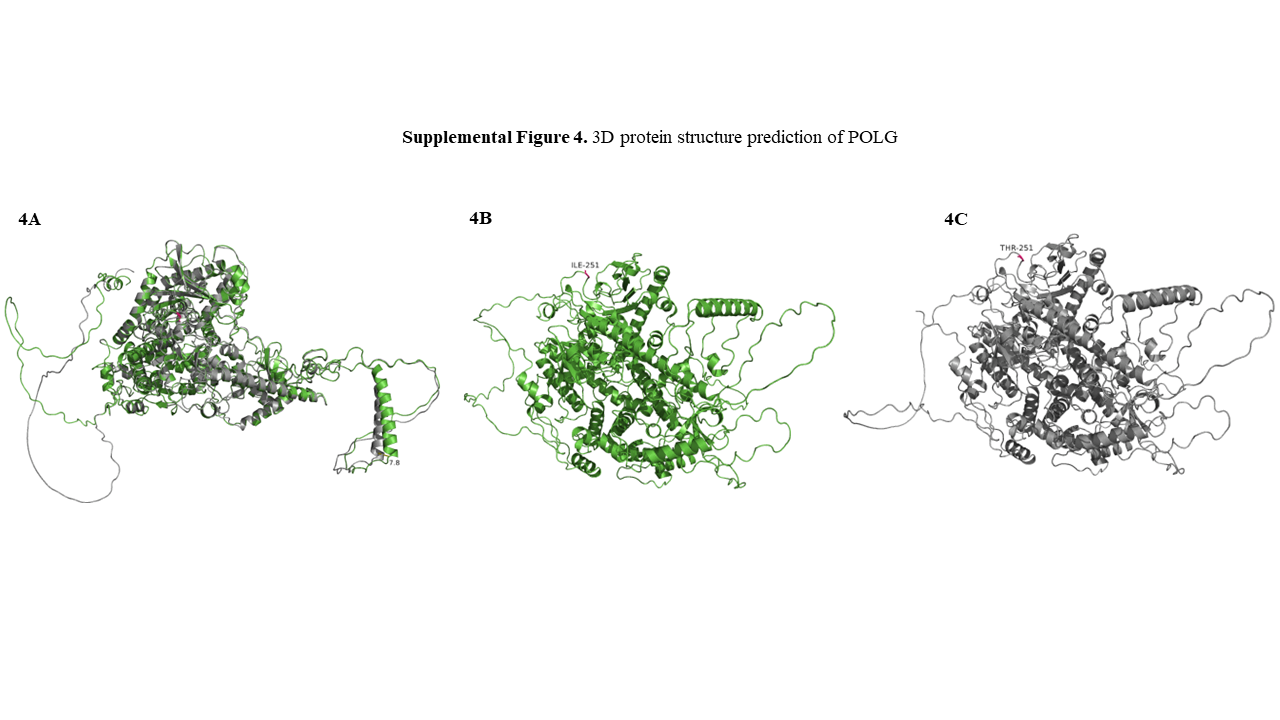

Supplement: Supplementary file 4 — Additional file 4. [file 13023_2024_3437_MOESM4_ESM.tiff]

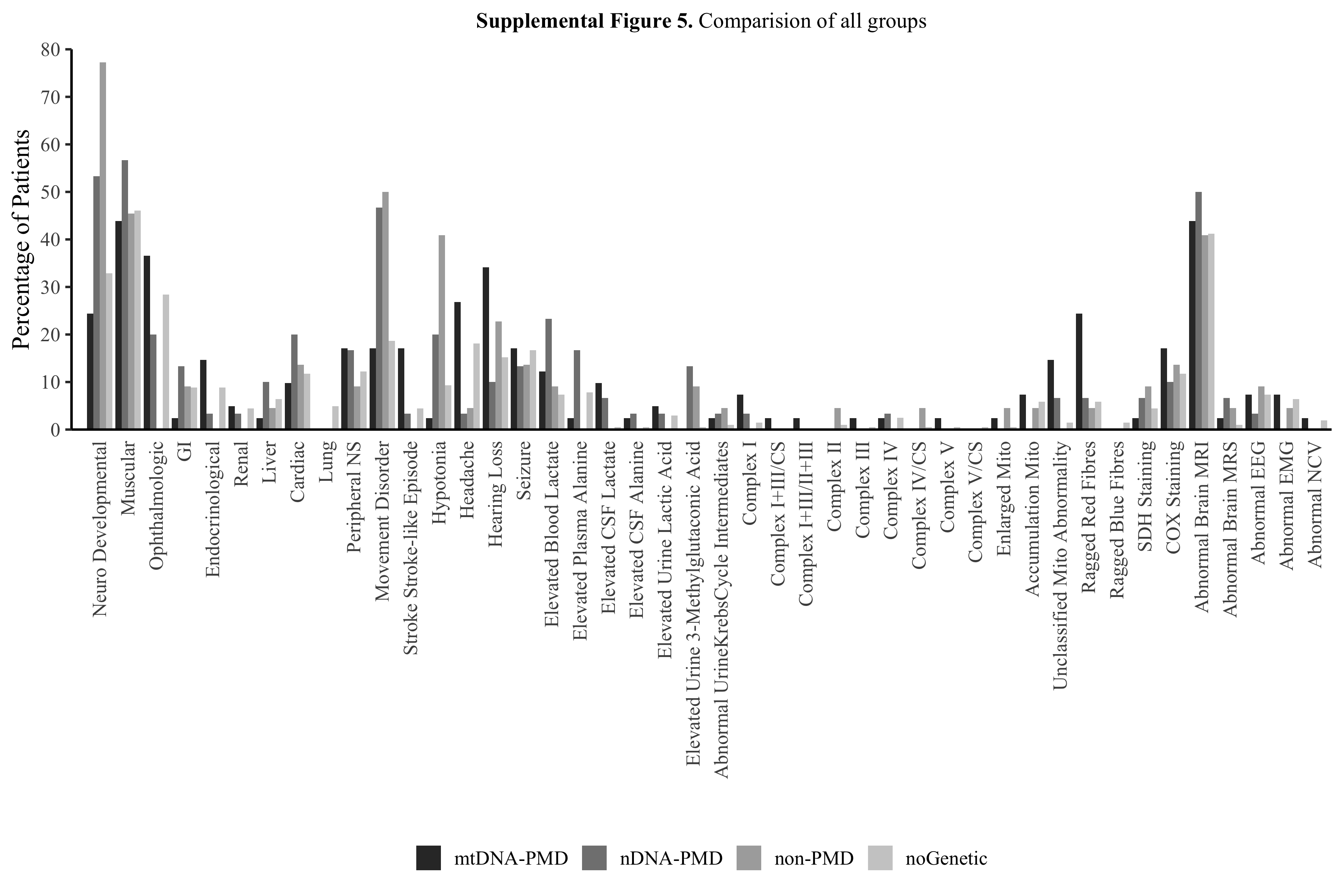

Supplement: Supplementary file 5 — Additional file 5. [file 13023_2024_3437_MOESM5_ESM.tiff]

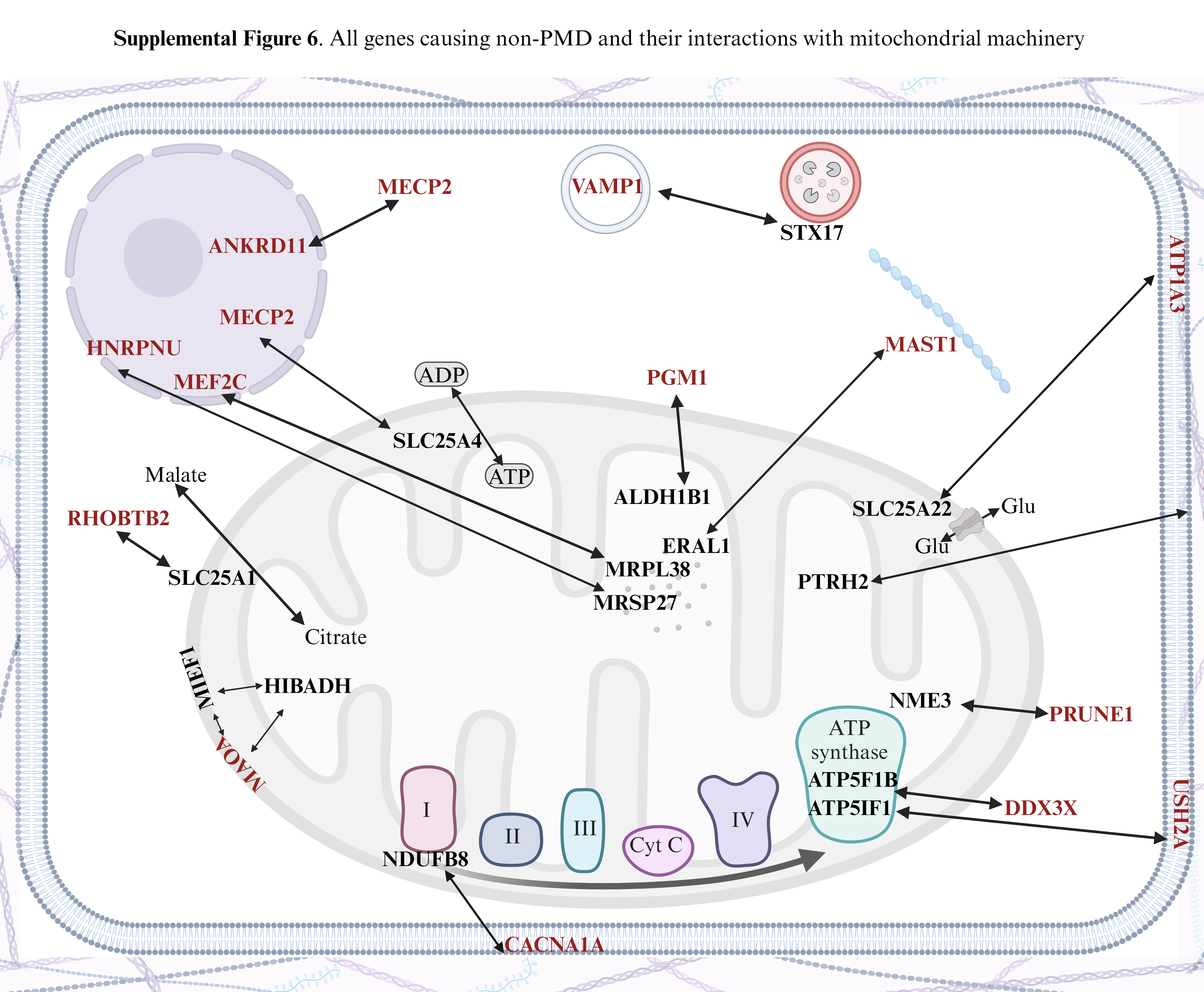

Supplement: Supplementary file 6 — Additional file 6. [file 13023_2024_3437_MOESM6_ESM.tiff]
