# Supplementary material for: Genetic landscape of primary mitochondrial diseases in children and adults using molecular genetics and genomic investigations of mitochondrial and nuclear genome
Source: Orphanet J Rare Dis. 2024 Nov 12;19:424. doi: 10.1186/s13023-024-03437-x (PMC11555972; doi:10.1186/s13023-024-03437-x)
Supplement: Supplementary file 7 — Additional file 7. [file 13023_2024_3437_MOESM7_ESM.docx]

**Supplemental Table 1**. Phenotypes, neuroimaging, biochemical features and genotypes of individuals with mtDNA-PMD

| **Number/study ID/diagnosis/sex/age** | **Clinical features (age of onset)** | **Neuroimaging** | **Other abnormal investigations** | **Molecular genetic and genomic test result/ heteroplasmy/ test type/ inheritance pattern** |
| --- | --- | --- | --- | --- |
| 1/Mito103/MELAS/F/15yrs | Headache, blurred vision, seizure, bilateral sensorineural hearing loss, verbal delay, stroke-like episode (1yrs) | Brain MRI=right parietal and left parieto-occipital WM signal | Blood lactate=4.3 | m.3243A>G in *MT-TL1*/ 71% heteroplasmy/ MELAS/MERRF/NARP panel, blood/ unknown |
| 2/Mito374/MELAS/F/21yrs | Low weight, short stature, cerebellar ataxia, GTC seizures, stroke-like episode (18yrs) | Brain MRI=  Small left occipital cortical focus of altered signal, likely representing early subacute ischemic injury | UOA lactic acid=5516/ CSF lactate=9.5/ ECG=sinus tachycardia, borderline rightward axis | m.13513G>A, p.Asp393Asn in *MT-ND4*/ 78% heteroplasmy/ MGS, urine/ unknown |
| 3/Mito058/MELAS/F/43yrs | Stroke-like episode, myalgia, migraines, left hearing loss (42yrs) | Brain MRI=T2 hyperintensities in subcortical WM/ brain MRS= lactate peak | NR | m.3243A>G in *MT-TL1*/ 11% heteroplasmy/ MELAS/MERRF/NARP panel, blood/ maternal |
| 4/Mito102/MELAS/F/46yrs | Stroke-like episodes, migraines, blurry vision (42yrs) | Brain MRI=abnormal T2 signal in temporal occipital and temporal parieto-occipital region, prominent cerebral sulci, subcortical WM hyperintensities in cerebral hemisphere/ EEG=intermittent sharp waves | Blood lactate=2.6/ CSF lactate=6.9/ EMG=prolonged PR interval | m.3243A>G in *MT-TL1*/ 31% heteroplasmy/ MGS, buccal/ unknown |
| 5/Mito104/MELAS/F/35yrs | Stroke-like episodes, bilateral sensorineural hearing loss, intellectual disability, seizures, tremor (28) | Brain MRI= T2 hyperintensity in left parietal and temporal lobes/ EEG=electrographic seizures, low-amplitude beta activity | NR | m.3243A>G in *MT-TL1*/ 77% heteroplasmy/ Mitochondrial genome panel, urine/ unknown |
| 6/Mito105/MELAS/F/43yrs | Seizure, lactic acidosis, stroke-like episodes (36yrs) | Brain MRI=hyperintensity in left temporal, anterior temporal, and right parietal convexity/ EEG= parietal and temporal electrographic seizures | Blood lactate=4.7/ UOA lactic acid/ CSF lactate=4.6 | m.3243A>G in *MT-TL1*/ 56% heteroplasmy/ Mitochondrial genome panel, urine/ unknown |
| 7/Mito118/MELAS/F/47yrs | Stroke-like episodes, hearing loss, headache, impaired balance, neuropathy, DM (15yrs) | Brain MRI= cortical/subcortical T2-hyperintensity with gyral swelling in left temporal lobe and left lateral occipital lobe with T1-hypointensity,  T2 hyperintensities within the supratentorial WM likely related to small vessel ischemic changes, volume loss noted | PAA alanine=618 | m.3243A>G in *MT-TL1*/ 56% heteroplasmy/ MELAS/MERRF/NARP panel, urine/ maternal |
| 8/Mito366/ MIDD/M/66yrs | DM, deafness (44yrs) | Brain PET= hypometabolism in right mid temporal opercular region | NR | m.3243A>G in *MT-TL1*/ 61% heteroplasmy/ MELAS mitochondrial screening, urine/ maternal |
| 9/Mito145/ MIDD/F/37yrs | Hearing loss, amblyopia, DM, deafness, left ventricular hypertrophy (37yrs) | Brain MRI=N | NR | m.3243A>G in *MT-TL1*/ 93% heteroplasmy/ Mitochondrial genome panel, urine/ unknown |
| 10/Mito14/MIDD/F/30yrs | Hearing loss, fatigue (5yrs) | Brain MRI=N | NR | m.3243A>G in *MT-TL1*/ 95% heteroplasmy/ Mitochondrial genome panel, urine/ maternal |
| 11/Mito055/Leigh syndrome/F/6mo/deceased | Failure to thrive, feeding difficulty, developmental delay, hypotonia, seizures, ventricular hypertrophy (neonatal) | Neuropathology autopsy=cerebral atrophy, cystic cavitary degeneration in corpus striatum, thalamus, brainstem tectal structures, and substantia nigra | Muscle biopsy=NADH cytochrome C reductase 0.39 (control 0.53-2.72)/ ECHO=left ventricular hypertrophy | m.14487T>C, p.Met63Val in *MT-ND6*/ homoplasmic/ MGS, blood/unknown |
| 12/Mito221/mtDNA Leigh syndrome spectrum/F/7yrs | Headache, myalgia, weakness, fatigue, paresthesia, bicuspid aortic valve (NB) | NR | NR | m.13513G>A, p.Asp393Asn in *MT-ND5/* 74% heteroplasmy/ MGS muscle/ unknown |
| 13/Mito119/mtDNA-associated Leigh syndrome spectrum/F/48yrs^1^ | Muscle spasms, muscle weakness, fatigue, headache, myalgia (39 yrs) | NR | NR | m.14487T>C in *MT-ND6*/ 84% heteroplasmy/ Mitochondrial screening, buccal/ unknown |
| 14/Mito120/ mtDNA-associated Leigh syndrome spectrum /F/12yrs^1^ | Developmental delay (4yrs) | NR | NR | m.14487T>C in *MT-ND6*/ 25% heteroplasmy/ Mitochondrial screening, blood, buccal/ maternal |
| 15/Mito131/ mtDNA-associated Leigh syndrome spectrum /F/63yrs | Polymyositis, lupus, muscle spasms, hearing loss (25yrs) | NR | NR | m.14487T>C in *MT-ND6/* 33% heteroplasmy/ Mitochondrial screening, urine/ maternal |
| 16/Mito214/Kearns-Sayre Syndrome/M/15yrs | Ophthalmoplegia, ptosis, sensorineural hearing loss, incomplete heart block, renal tubulopathy, intellectual disability, motor impairment | Brain MRI= signal abnormality in cerebral and cerebellar WM, basal ganglia, thalami, and brainstem,  cerebral atrophy/ brain MRS=lactate peak | Kidney US=interval growth of left kidney/ UOA alanine=943 | 6.8kb deletion/ ~7% heteroplasmy/ Mitochondrial deletion southern blot, blood/ unknown |
| 17/Mito001/CPEO/F/72yrs | Hearing loss, ophthalmoplegia, headache, ptosis (52yrs) | Brain MRI=N | NR | m.6342_14004del (7.6kb) in *MT-CO1* to *MT-ND5*/ 20% heteroplasmy/ Mitochondrial screening, muscle/ unknown |
| 18/Mito002/CPEO/M/67yrs | Ptosis, external ophthalmoplegia, myopathy, rhabdomyolysis, chronic pain, fatigue, sensorineural hearing loss, tinnitus, myoglobinuria, acute renal failure (41yrs) | Brain MRI=N | Muscle biopsy=accumulation of mitochondria | Mitochondrial screening, blood/ 7kb deletion/ 68% heteroplasmy/ muscle biopsy |
| 19/Mito008/CPEO/F/59yrs | Ophthalmoparesis, bilateral ptosis, myalgia, progressive muscle weakness, fatigue, headaches (47yrs) | Brain MRI=T2 hyperintensities in supratentorial WM | Muscle biopsy=RRFs, NRDH cytochrome C reductase 0.39 (control 0.53-2.72)/ EMG=non-disfigurative myopathy/ NCV=sensory neuropathy | 7.4kb m.8649_16086del in *MT-ATP6* to *MT-CYB/* 25% heteroplasmy/ MGS, muscle/ presumed de novo |
| 20/Mito095/CPEO/M/26yrs | Progressive double vision, ptosis, reduced muscle bulk (15yrs) | NR | NR | 8.2kb deletion in CPEO region/ 30% heteroplasmy/ MGS, muscle/ unknown |
| 21/Mito096/CPEO/M/37yrs | External ophthalmoplegia, ptosis, fatigue, migraines (NR) | NR | Muscle biopsy=RRFs, accumulation of mitochondria/ EMG=non-disfigured myopathy | m.12016_14845del (2.8kb) in *MT-ND4* to *MT-CYB*/ 40-50% heteroplasmy/ Mitochondrial genome panel, muscle/ unknown |
| 22/Mito111/CPEO/F/71yrs | Weakness, pain, fatigue, ptosis, diarrhea, thrombosis, external ophthalmoplegia (67yrs) | Brain MRI=non-occlusive thrombus in transverse and superior sagittal sinuses, T2 hyperintensity in cerebral WM, intracranial arterial vascular flow voids | Muscle biopsy=RRFs | 5kb deletion spanning *MT-ATP6* to *MT-ND5*/ NR/ mitochondrial deletion southern blot, muscle/ unknown |
| 23/Mito112/CPEO/F/28yrs | Bilateral ptosis, external ophthalmoplegia (18yrs) | NR | NR | m.11037_15281del (4.2kb) in *MT-ND4* to  *MT-CYB*/ 25% heteroplasmy/ Mitochondrial screening, urine/ unknown |
| 24/Mito116/CPEO/M/48yrs | Progressive ophthalmoplegia, diplopia (42yrs) | NR | Muscle biopsy=RRFs | m.6226_11232del (5.7kb) in *MT-CO1* to *MT-ND4*/ 20% heteroplasmy/ MGS muscle/ unknown |
| 25/Mito117/CPEO/M/64yrs | Myalgia, fatigue, hearing loss, liver cirrhosis (55yrs) | NR | NR | 5kb deletion spanning *MT-ATP6* to  *MT-ND5*/ NR/ Mitochondrial tissue biopsy/ unknown |
| 26/Mito148/CPEO/M/42yrs | Ophthalmoplegia, left ptosis, muscle pain with exercise, depression, retinitis pigmentosa, OCD, autistic features (teenage years) | Brain MRI=N | Muscle biopsy=RRFs | m.11275_15514del (4.2kb) spanning *MT-ND4* to  *MT-CYB*/ 50% heteroplasmy/ Mitochondrial deletion analysis, muscle/ unknown |
| 27/Mito195/CPEO/F/51yrs | External ophthalmoplegia, diplopia, ptosis, reduced oculomotility (47yrs) | NR | Muscle biopsy=RRFs | m.8482_13447del (4965kb) spanning MT-ATP8 to MT-ND4/ 45% heteroplasmy/ Mitochondrial screening, muscle/ unknown |
| 28/Mito196/CPEO/M/53yrs | Cranial nerve dysfunction, dysautonomia, inflammatory demyelinating polyneuropathy, cryoglobulinemia, seizures, porphyria cutanea tarda, impaired hearing, myopathy, carpal tunnel syndrome (NR) | NR | Muscle biopsy=COX-negative fibers, reduced SDH staining | Multiple mitochondrial DNA deletions  (6-10.5 kb) in KSS region/ 20-30% heteroplasmy/mitochondrial genome sequencing, muscle/ unknown |
| 29/Mito268/CPEO/64yrs | Ophthalmoplegia, heart murmur, ptosis, DM, cataracts (40yrs) | NR | Muscle biopsy=RRFs | m.11038delA, p.Lys93Asnfs*7 in *MT-ND4*/ 12% heteroplasmy/ Mitochondrial screening, urine/ unknown |
| 30/Mito282/CPEO/61yrs/deceased | Ptosis, digestive problems, failure to thrive, hypothyroidism, exercise intolerance (NR) | Brain MRI=scattered foci of hyperintensity in supratentorial WM | Muscle biopsy=RBFs | m.9924_16073del (6149bp) in *MT-COIII* to *MT-*CYB/ 12% heteroplasmy/ Mitochondrial deletion southern blot, muscle/ unknown/ ES negative |
| 31/Mito316/CPEO plus/M/59yrs | Atrial fibrillation, transient ischemic attack, cluster headaches, myalgia, ophthalmoplegia, ptosis (56yrs) | Brain MRI=non-specific supratentorial WM foci | Muscle biopsy=COX-negative fibers, reduced SDH staining | m.9761_16067del (12.9kb)/ 24% heteroplasmy/ MGS, muscle/ unknown |
| 32/Mito348/CPEO/F/26yrs | Headaches, ophthalmoparesis, bilateral ptosis, diplopia, myasthenia gravis (22yrs) | Brain MRI=N | Muscle biopsy=accumulation of mitochondria, large mitochondria/ EMG=myopathic changes in right upper extremity | m.8469_13446del (5kb) in *MT-ATP8 to MT-ND5/* 20-30% heteroplasmy/ Mitochondrial genome deletion/duplication analysis, muscle/ unknown |
| 33/Mito353/Multi-systemic syndrome/M/31yrs | Hypertension, neuropathy, right optic neuritis, impaired digestion, kidney disease, nephritis, hearing loss (23yrs) | Brain MRI=progression of optic atrophy | Kidney US=increased echotexture bilaterally/ ECG=prominent T waves in precordial leads/ CSF lactate=3.4/ muscle biopsy=RRFs | m.13513G>A, p.Asp393Asn in *MT-ND5/* 63% heteroplasmy/ MGS, muscle/ unknown |
| 34/Mito383/Multi-systemic syndrome/F/75yrs | Ischemic heart disease, hypertrophic cardiomyopathy, central areolar choroidal dystrophy, sensorineural hearing loss (69yrs) | Brain MRI=WM hyperintense lesions in supratentorial parenchyma | Muscle biopsy=RRFs | m.3243A>G in *MT-TL1*/ 59% heteroplasmy/ MGS, muscle/ unknown |
| 35/Mito318/Multi-systemic syndrome/F/39yrs | Stroke-like episodes, hearing loss, DM, ketoacidosis (35yrs) | Brain MRI=cerebral and cerebellar atrophy, cerebellar peduncle volume loss, T2 hyperintense foci in subcortical WM/  brain MRS=N | Plasma 3-MGA=515 | m.8681_8682del, p.Leu52Hisfs*12 in *MT-ATP6/* 41% heteroplasmy/ MGS, urine/ unknown |
| 36/Mito003/LHON/M/62yrs/deceased | White eyesight changes, impaired vision (34yrs) | NR | NR | m.11778G>A, p.Arg340His in *MT-ND4/* homoplasmic/ LHON analysis, blood/ maternal |
| 37/Mito101/LHON/F/58yrs | Bilateral vision impairment, colour blindness, astigmatism, non-alcoholic steatohepatitis (53yrs) | Brain MRI=subcortical T2 focal hyperintensities in supratentorial WM | ECG=left ventricular hypertrophy | m.14484T>C in *MT-ND6/* homoplasmic/ LHON analysis, blood/ maternal |
| 38/Mito004/MERRF/M/55yrs/deceased | Muscle weakness, hearing loss, progressive myopathy, chronic fatigue (44yrs) | NR | PAA alanine=591/ blood lactate=3.0/ muscle biopsy=RRFs | m.8344A>G in *MT-TK*/ homoplasmic/ MGS, muscle/ maternal |
| 39/Mito147/MERRF/F/51yrs | Headache, diplopia, weakness, tingling, cognitive dysfunction, myoclonus (20yrs) | Brain MRI= T2 hyperintensities in supratentorial WM | Muscle biopsy=RRFs/ ECG=sinus rhythm, nonspecific intraventricular conduction delay, ST elevation | m.8344A>G in *MT-TK*/ 38% heteroplasmy/ Mitochondrial genome panel, urine/ maternal |
| 40/Mito229/NARP/F/77yrs/deceased | Muscle weakness, peripheral neuropathy, ataxia, seizures, retinitis pigmentosa, optic atrophy, learning disability (59yrs) | Brain MRI=cerebral and cerebellar atrophy | NR | m.8993T>G in *MT-ATP6*/ 48% heteroplasmy/ Mitochondrial genome panel, urine, blood/ unknown |
| 41/Mito397/Mitochondrial complex I deficiency/M/46yrs | Hypopituitarism, muscle fatigue, muscle weakness, ataxia, dyspnea, chest tightness (40yrs) | NR | NR | m.3359_3360del, p.Ala18Valfs*11 in *MT-ND1*/ 4% heteroplasmy/ MGS, urine/ maternal |

^1^Superscript indicates family number

**Abbreviations**: COX=cytochrome c oxidase; CPEO=Chronic progressive external ophthalmoplegia; DM=diabetes mellitus; ECHO=echocardiography; EEG=electroencephalography; ES=exome sequencing; GDD=global developmental delay; GERD= gastroesophageal reflux disease; HEMZ= hemizygous; HMZ= homozygous; HTZ= heterozygous; LHON=Leber hereditary optic neuropathy; MELAS=Mitochondrial Encephalopathy, Lactic Acidosis, and Stroke-like episodes; MERRF=Myoclonus epilepsy with ragged-red fibers; MIDD=Maternally inherited diabetes and deafness; MGS=mitochondrial genome sequencing; mo(s)=month(s); mtDNA=mitochondrial DNA; MRI=magnetic resonance imaging; NARP=Neuropathy, ataxia, and retinitis pigmentosa; NR=not reported; PAA= plasma amino acids; PVWM= periventricular won; RBFs=ragged blue fibers; RRFs=ragged red fibers; UOA= urine organic acid; ES=whole exome sequencing; WM= white matter; yr(s)=year(s)

**Reference Ranges:** PAA alanine adult: 180-600 umol/L; PAA alanine child: 13-48 umol/L; blood lactate child: 0.5-2.2 mmol/L; UOA 3-MGA adult: 15-87; UOA lactic acid adult: <400 mmol/mol creatinine; plasma 3-MGA adult=; plasma 3-MGA child= 103-384 nmol/L; CSF lactate adult: 1.1-2.4 mmol/L; CSF alanine: <48 mmol/L

**Supplemental Table 2**. Phenotypes, neuroimaging, biochemical features and genotypes of individuals with nDNA-PMD

| **Number/study ID/diagnosis/sex/age** | **Clinical features (age of onset)** | **Neuroimaging** | **Other abnormal investigations** | **Molecular genetic and genomic test result/ test type/ inheritance pattern** |
| --- | --- | --- | --- | --- |
| 1/Mito035/Combined oxidative phosphorylation deficiency 12/F/18yrs | Failure to thrive, dysphagia, hypotonia, verbal and motor delay (2mo) | Brain MRI=abnormal T2 signal in subcortical WM in frontal and parietal lobes, cystic defects in genu and splenium, abnormal signal in thalami and cerebral aqueduct | Blood lactate=3.1/ CSF alanine=50 | CMP HTZ c.328G>A, p.Gly110Ser/  c.1004A>T, p.Asn335Ile in *EARS2*/ Mutation analysis of LBSL/ AR/ unknown |
| 2/Mito038/Pyruvate dehydrogenase E1-alpha deficiency/F/16yrs | Ptosis, bilateral sensorineural hearing loss, basal ganglia stroke, peripheral neuropathy, spasticity, hemiplegia, GDD, motor delay, learning disability (5yrs) | Brain MRI=bilateral globus pallidus infarcts, generalized brain atrophy/ Brain MRS=lactate peak | PAA alanine=762/ blood lactate=3.6/ CSF lactate=3.1 | HEMZ c.1046C>T, p.Ala349Val in *PDHA1*/ ES trio/ XLD maternal |
| 3/Mito033/Pyruvate dehydrogenase E1-alpha deficiency/M/19yrs | Weakness, slow speech, ptosis, severe ataxia, hypotonia, motor decompensation, poor balance, seizure (2yrs) | Brain MRI= bilateral globus pallidus and medullary lesions | PAA alanine=693/ blood lactate=2.4/ CSF lactate=7.6/ muscle biopsy= necrotic myofiber | HEMZ c.374T>C, p.Ile125Thr in *PDHA1/* PDH panel/ XLD maternal |
| 4/Mito041/3-methylglutaconic aciduria with deafness, encephalopathy, and leigh-like syndrome/M/15yrs | Microcephaly, developmental delay with regression, scoliosis, pectus excavatum, hypertonia, sensorineural hearing loss, right eye esotropia, vomiting, hypotonia, failure to thrive (5mo) | Brain MRI=T2 hyperintensity in frontal cortical and subcortical regions, diffuse cerebral, basal ganglia, cerebellar atrophy, ex vacuo dilatation of ventricles | UOA 3-MGA=39.6 | HMZ c.1822_1828+10del17insACCAACAGG in *SERAC1*/ Methylglutaconic aciduria nuclear gene panel/ AR/ unknown |
| 5/Mito092/Mitochondrial myopathy and ataxia/F/8yrs  Despite one VUS, we took as diagnosis | Brain atrophy, dysmetria, ataxia, apraxia, dysphagia, verbal delay, ventricular hypertrophy (4yrs) | Brain MRI=bilateral cerebellar atrophy, bilateral T2 hyperintensity in pons and superior cerebellar peduncles | NR | CMP HTZ c.354dup, p.Leu119Serfs*9 (maternal)/ c.296G>A, p.Gly99Glu (paternal) in *MSTO1*/ ES trio/ AR |
| 6/Mito098/3-methylglutaconic aciduria, type V/M/18yrs^1^ | Dilated cardiomyopathy, ataxia, microcephaly, myopathy, developmental delay | NR | UOA 3-MGA=56.5 | HMZ c.130-1G>C in *DNAJC19*/ Family testing/AR maternal, paternal |
| 7/Mito097/3-methylglutaconic aciduria, type V/F/27yrs^1^ | Muscle weakness, short stature, cardiomyopathy, developmental delay | Brain MRI=right axis deviation, nonspecific T abnormality | NR | HMZ c.130-1G>C in *DNAJC19*/ DNAJC19 gene sequencing/ AR/ maternal, paternal |
| 8/Mito202/3-methylglutaconic aciduria, type V/M/29yrs^1^ | Microcephaly, optic nerve hypoplasia, short stature, anemia, developmental delay, cerebellar dysfunction, ataxia, dilated cardiomyopathy (14yrs) | NR | Urine 3-MGA=19.2 | HMZ c.130-1G>C in *DNAJC19/* DNAJC19 gene sequencing/ AR maternal, paternal |
| 9/Mito100/3-methylglutaconic aciduria, type V/M/4yrs^2^ | Liver disease, cardiomyopathy, hypoglycemia, anemia, impaired digestion, GDD, growth retardation (8wks) | Brain MRI=left cerebellar tissue loss | UOA 3-MGA=25.8/ AST=139/ALT=184/ GGT=83/ | HMZ c.130-1G>C in *DNAJC19*/ ES trio/ AR maternal, paternal |
| 10/Mito130/3-methylglutaconic aciduria, type V/F/6mo^2^ | Dilated cardiomyopathy (2mo) | NR | Plasma 3-MGA=590 | HMZ c.130-1G>C in *DNAJC19*/ Cardiac Gene Panel/ AR maternal, paternal |
| 11/Mito205/3-methylglutaconic aciduria, type V/F/1yr/deceased | Dilated cardiomyopathy, heart failure, liver steatosis, developmental delay, failure to thrive (NB) | NR | UOA 3-MGA=13.7 | HMZ c.130-1G>C in *DNAJC19*/ Dilated Cardiomyopathy with Ataxia Syndrome panel/ AR, maternal, paternal |
| 12/Mito206/3-methylglutaconic aciduria, type V/M/40yrs/deceased | Muscle weakness, bilateral cataracts, progressive hearing loss, distal atrophy of upper and lower extremities (NA) | Brain CT=cerebellar atrophy | NR | HMZ c.130-1G>C in *DNAJC19*/ DNAJC19 gene sequencing/ AR maternal, paternal |
| 13/Mito125/Mitochondrial complex I deficiency, nuclear type 21/F/18yrs^3^ | Impaired balance and motor control (1yr) | Brain MRI=atrophy and T2 signal in cerebellar hemispheres and vermis, enlarged cisterna magna, cystic degeneration, ventral pons and medulla volume loss | NR | HMZ c.166G>A, p.Gly56Arg in *NUBPL*/ ES singleton/ AR maternal, paternal |
| 14/Mito126/Mitochondrial complex I deficiency, nuclear type 21/19yrs^3^ | Headache, constipation, cognitive dysfunction, impaired movement and coordination (2yr) | Brain MRI= atrophy and increased T2 signal in cerebral peduncles and ventral pons, cerebellar vermis and cerebellum atrophy, ex vacuo dilation of 4th ventricle, cerebellopontine angle cistern and cisterna magna | NR | HMZ c.166G>A, p.Gly56Arg in *NUBPL*/ ES singleton/ AR maternal, paternal |
| 15/Mito132/Mitochondrial DNA depletion syndrome 5/M/3yrs | Hypotonia, GDD, choreoathetosis, truncal ataxia (NB) | Brain MRI=N | NR | CMP HTZ c.998A>G, p.Asp333Gly (maternal)/  c.(271+1_272-1)(534+1_535-1)del (paternal) in *SUCLA2*/ ES trio, CNV analysis/ AR |
| 16/Mito133/Ataxia, early-onset, with oculomotor apraxia and hypoalbuminemia/M/9yrs^4^ | Peripheral neuropathy, dysmetria, ocular motor apraxia (7yrs) | Brain MRI= cerebellar hemisphere and vermis volume loss | NR | HMZ c.(483+1_484-1)(543+1_544-1)del, p.Glu162_Gln181del in *APTX*/ ES singleton/ AR, maternal, paternal |
| 17/Mito134/Ataxia, early-onset, with oculomotor apraxia and hypoalbuminemia/F/7yrs^4^ | Ataxia, ocular motor apraxia (6yrs) | Brain MRI= cerebellar hemisphere and vermis volume loss | NR | c.(483+1_484-1)(543+1_544-1)del, p.Glu162_Gln181del in *APTX*/ Family testing/AR maternal, paternal |
| 18/Mito135/Ataxia, early-onset, with oculomotor apraxia and hypoalbuminemia/F/15yrs^4^ | Dysmetria, ocular motor apraxia, cerebellar and vermis hypoplasia, dystonia, ataxia, developmental delay (14yrs) | Brain MRI=progressive cerebellar and vermis hypoplasia | NR | HMZ c.(483+1_484-1)(543+1_544-1)del, p.Glu162_Gln181del in *APTX*/ ES trio/AR maternal, paternal |
| 19/Mito194/Harel-Yoon syndrome/F/4wk/died | Hypotonia, encephalopathy, bilateral corneal opacities, hypertrophy (NB) | EEG=bilateral multifocal sharp waves/ Brain MRI=bilateral infarct on thalami, intraventricular hemorrhage in occipital horns, subdural hemorrhage posteriorly on falx and posterior cerebellum | Blood lactate=12.3/ blood anion gap=17 | HTZ chr1:g.1392414_1459894dup in *ATAD3A*/ ES trio/ AD de novo |
| 20/Mito217/Multiple mitochondrial dysfunctions syndrome 6/F/6yrs | Microcephaly, muscle twitch, developmental delay (NB) | NR | Blood lactate=2.7 | CMP HTZ c. 788_791delCTTT, p.Ser263Tyrfs*23 (paternal)/  c.1321A>G, p. Arg441Gly (maternal) in *PMPCB*/ ES duo/ AR |
| 21/Mito256/Encephalopathy, progressive, early-onset, with brain edema and/or leukoencephalopathy/F/1yrs/deceased | Neurological regression, comatose, cardiac arrest, encephalopathy, ptosis, esotropia, hypotonia, microcephaly, muscle weakness (7mo) | NR | NR | HMZ c.177C>A, p.Tyr59* in *NAXE*/ ES trio/ maternal, paternal |
| 22/Mito375/Combined oxidative phosphorylation deficiency 11/M/2yrs  Despite one VUS, we took as diagnosis | Hypertension, seizure, bilateral sensorineural hearing loss, echogenic kidneys (3mo) | Brain MRI=N/ Brain MRS=lactate peak | Blood lactate=2.7/ muscle biopsy=RRFs, COX-negative fibers, structurally abnormal mitochondria | CMP HTZ c.713A>G, p.Asn238Ser (maternal), c.761A>G, p.His254Agr (paternal) in *RMND1*/ ES trio/ AR |
| 23/Mito016/Mitochondrial complex I deficiency, nuclear type 4/F/38yrs | Muscle pain, muscle weakness, muscle spasms, leg limp, myopathy, progressive fatigue, optic atrophy, nocturnal hypoventilation, paresthesia of fingertips and toes, constipation, urinary retention, neurogenic bladder (14yrs) | Brain MRI= symmetrical T2 signal abnormalities in putamina and caudate nuclei | PAA alanine=1000 | HMZ c.640G>A, p.Glu214Lys in *NDUFV1*/ ES singleton/ AR maternal, paternal |
| 24/Mito017/Combined oxidative phosphorylation deficiency 15/M/36yrs | Seizures, ptosis, impaired vision, dysarthria, splenomegaly, developmental delay with regression, optic neuropathy (28yrs) | Brain MRI= lesions, bilateral hyperintensities on globus pallidus, medial thalami, brainstem and PVWM of right frontal lobe | PAA alanine=762 | HMZ c.626C>T, p.Ser209Leu in *MTFMT*/ ES trio/ AR maternal, paternal |
| 25/Mito032/Progressive external ophthalmoplegia/F/21yrs | Ophthalmoparesis, ptosis, skeletal myopathy, esotropia, facial muscle weakness (3yrs) | Brain MRI=N | Muscle biopsy=RRFs, cytochrome oxidase abnormality | HTZ c.817T>C, p.Phe273Leu in *POLG*/ ES trio/ AD de novo |
| 26/Mito129/Mitochondrial recessive ataxia syndrome/F/50yrs | Progressive polyneuropathy, neuropathic pain, fatigue, spasms, light-headedness, hypothyroidism, anxiety (35yrs) | NA | NA | c.1760C>T, p.Pro587Leu/  c.752C>T, p.Thr251Ile in *POLG*/ES plus/ AR, unknown |
| 27/Mito106/Combined oxidative phosphorylation deficiency 6/M/28yrs | Peripheral neuropathy muscle twitch, ataxia, weakness (7mo) | NR | Blood lactate=3.7 | HEMZ c.845C>G, p.Thr282Arg in *AIFM1*/ AIFM1 gene sequencing/ XLR maternal |
| 28/Mito109/Progressive external ophthalmoplegia with mitochondrial DNA deletions 5/M/51yrs | Myopathy, muscle pain, muscle weakness, progressive external ophthalmoplegia, DM (49yrs) | NR | Muscle biopsy=large mitochondria, reduced COX staining | HTZ c.253_255del, p.Glu85del in *RRM2B*/ ES singleton/ AD/ unknown |
| 29/Mito123/Combined Oxidative Phosphorylation Deficiency 33/F/62yrs | Chronic progressive external ophthalmoplegia, congenital exotropia, left amblyopia, bilateral ptosis, myalgia, weakness, weight loss, fatigue (58yrs) | NR | NR | HMZ c.377G>A, p.Gly126Glu in *C1QBP*/ ES trio/ AR maternal, paternal |
| 30/Mito127/Combined oxidative phosphorylation deficiency 1/33yrs | Encephalopathy, seizures, liver dysfunction, splenomegaly, hepatic dysfunction, cognitive dysfunction, unstable gait (early childhood) | NR | Muscle biopsy=reduced cytochrome C oxidase activity | CMP HTZ c.910A>G, p.Lys304Glu/  c.1297_1300delGACA, p.Asp433Lysfs*20 in *GFM1*/ Nuclear encoded mitochondriopathies panel/ AR, unknown |

^1-4^ Superscript indicates family number

**Abbreviations**: AD= autosomal dominant; ALT=alanine transaminase; AR=autosomal recessive; AST=aspartate aminotransferase; COX=cytochrome C oxidase; DM=diabetes mellitus; EEG=electroencephalography; GDD=global developmental delay; GGT= gamma-glutamyl transpeptidase; hrs=hours; HEMZ= hemizygous; HMZ= homozygous; HTZ= heterozygous; mo=months; MRI=magnetic resonance imaging; NB=newborn; NR=not reported; PAA= plasma amino acids; PVWM= periventricular won; RRFs=ragged red fibers; SDH=succinate dehydrogenase; UOA= urine organic acid; ES=whole exome sequencing; WM= white matter; yrs=years

**Reference Ranges:** PAA alanine adult: 180-600 umol/L; PAA alanine child: 13-48 umol/L; blood lactate child: 0.5-2.2 mmol/L; UOA 3-MGA adult: 15-87; UOA lactic acic adult: <400 mmol/mol creatinine; plasma 3-MGA child= 103-384 nmol/L; CSF lactate adult: 1.1-2.4 mmol/L; CSF alanine: <48 mmol/L

**Supplemental Table 3**. Phenotypes, neuroimaging, biochemical features, and genotypes of individuals with non-PMD

| **Number/study ID/diagnosis/sex/age** | **Clinical features (age of onset)** | **Neuroimaging** | **Other abnormal investigations** | **Molecular genetic and genomic test result/ test type/ inheritance pattern** |
| --- | --- | --- | --- | --- |
| 1/Mito044/Rett syndrome/M/13yrs/deceased^1^ | GDD, cranial neuropathy, bibrachial neuropathy, seizures, bilateral esotropia, choreoathetosis (NR) | Brain MRI=N | Blood lactate=3.1 | HTZ c.942delC, p.Ile315Serfs*18 in *MECP2/* MECP2 gene sequencing/ XLD maternal |
| 2/Mito047/ Cerebellar ataxia, areflexia, pes cavus, optic atrophy, and sensorineural hearing loss syndrome/F/13yrs^2^ | Hypotonia, dystonia, parkinsonism, motor and speech delay, developmental regression, chorea-athetosis, areflexia (4yrs) | Brain MRI=N | Muscle biopsy=succinate dehydrogenase 9.0 (control 18.6-47.0) | HTZ c.2267G>A, p.Arg756His in *ATP1A3*/ ES trio/ AD de novo |
| 3/Mito052/Neurodevelopmental disorder with hypotonia, stereotypic hand movements, and impaired language/M/11yrs^3^ | GDD, febrile seizures, intellectual disability, autism, hypotonia, dyskinesia, myopathy, bruxism (10yrs) | Brain MRI=delayed myelination | Muscle biopsy=accumulation of mitochondria, large mitochondria | HTZ c.51_54del, p.Gln18 in *MEF2C*/ ES trio/ AD, de novo |
| 4/Mito066/Bethlem myopathy 2/F/13yrs^4^ | Hypertrophic scars, kyphosis, macrocephaly, dysmorphic features, umbilical hernia, hypotonia (NB) | NR | Muscle biopsy=accumulation of mitochondria | HTZ c.8319+1G>T in *COL12A1*/ Sherbrooke Muscle Disorders Panel/ AD unknown |
| 5/Mito128/Intellectual developmental disorder, X-linked syndromic, Billuart type/M/10yrs^5^ | Macrocephaly, seizures, hypotonia, autism, GDD (NB) | NR | NR | HEMZ c.1421-2A>G in *OPHN1*/ ES trio/ XLR maternal |
| 6/Mito154/KGB syndrome/M/4yrs^6^ | Dysmorphic features (thin upper lip, less defined philtrum, upturned nose, high arched palate), acute otitis media, motor delay, language dysfunction (1yrs) | Brain MRI=T2 hyperintensity in left PVWM | NR | HTZ c.7790A>G, p.Lys2597Arg in *ANKRD11*/ ES trio/ AD de novo |
| 7/Mito169/Limb-girdle muscular dystrophy type 2S/M/15yrs^7^ | Rhabdomyolysis, cognitive dysfunction, choreoathetosis, tremor, impaired balance, left ventricular hypertrophy, cataracts (10yrs) | NR | ECG=left ventricular hypertrophy | HMZ c.1287+5G>A in *TRAPPC11*/ TRAPPC11 gene sequencing/ AR unknown |
| 8/Mito222/Coffin-siris syndrome/F/1.5yrs/deceased^8^ | Pulmonary stenosis, dysmorphic features (short epicanthal folds, coarse hair), developmental delay (7wks) | NR | NR | 2.98 Mb deletion, chr17:37894463-40875639del encompassing 139 RefSeq genes/ microarray/ unknown |
| 9/Mito264/Intellectual developmental disorder, X-linked syndromic, Snijders Blok type/F/7yrs^9^ | GDD, failure to thrive, dysmorphic features (beaked nose, hypoplastic ala nasi, low nasal bridge), ASD, sinus arrythmia (5mo) | Brian MRI= cerebral parenchymal volume loss, thinning of PVWM | UOA 3-MGA=9.5/ blood lactate=2.5 | HTZ c.1012T>G, p.Leu338Val in *DDX3X*/ ES trio/ AD de novo |
| 10/Mito267/Developmental non-epileptic encephalopathy 42/M/6yrs^10^ | Developmental verbal and motor delay, infantile spasms, sensorineural hearing loss, hypotonia, hypertonia, dystonia, ASD, ear helices (NB) | Brain MRI=enlarged lateral ventricles, prominent cerebral sulci, thin corpus callosum, periventricular T2 hyperintensity, stable scaphocephaly/ EEG=slow background rhythm with overlying fast activity | NR | HTZ c.985G>A, p.Asp329Asn in *CACNA1A*/ ES trio/ AD de novo |
| 11/Mito299/Neurodevelopmental disorder with microcephaly, hypotonia, and variable brain anomalies/M/1yr/deceased^11^ | Spasms, hypoxia, hypotonia, neuroregression (NB) | Brain MRI=diffusion restriction in thalami, medial globus pallidus and brainstem, T2 hyperintensity in PVWM, cerebral atrophy, ventricular enlargement | NR | HMZ c.521-2A>G in *PRUNE1*/ ES trio/ AR maternal, paternal |
| 12/Mito300/Mega-corpus-callosum syndrome with cerebellar hypoplasia and cortical malformations/F/1.5yrs/deceased^12^ | Infantile spasms, hypsarrhythmia, GDD with regression, dysmorphic features (cushinoid face, small nose with depressed nasal bridge, prominent cupid lip) (5mo) | Brain MRI= PVWM volume loss/ Brain MRS= diminished lactate peak | NR | HTZ c.994C>T, p.Arg332Cys in *MAST1*/ ES trio/ AD de novo |
| 13/Mito378/Neurodevelopmental disorder/M/3mo/deceased^13^ | GDD, motor delay, congenital esotropia, hypotonia, truncal ataxia (3mo) | Brain MRI=N | NR | HTZ c.4045C>G, p.Arg1349Gly in *CACNA1A*/ Blueprint ataxia panel/ AD de novo |
| 14/Mito389/Usher Syndrome type 2A/F/14yrs^14^ | Sensorineural hearing loss, verbal delay (9yrs) | NR | NR | HMZ c.5776+1G>A in *USH2A*/ USH2A gene sequencing/ AR unknown |
| 15/Mito009/Neuropathy, recurrent, with pressure palsies/M/47yrs/deceased^15^ | Numbness, muscle weakness, incoordination, deteriorating vision, tremors, joint pain, neurogenic bladder, dysphagia, dysarthria, hearing loss (17yrs) | Brain MRI=N | Muscle biopsy=RRFs/ EMG=demyelinating polyneuropathy affecting sensory and motor nerve fibers, ulnar neuropathies | 1.45 Mb deletion, chr17:13970367-15552448delin *PMP22, CDRT15, HS3DT3B1, COX10, LINCO2096, MIR4731, SNORA174, TVP23C,* *CDRT4, TEKT3*/ microarray/ AD maternal |
| 16/Mito070/Brunner Syndrome/M/22yrs^16^ | Diarrhea, intellectual disability, sensorimotor developmental delay, hypotonia, tremor, abnormal behavior (NA) | NR | NR | HEMZ c.145G>A, p.Gly49Arg in *MAOA*/ ES trio/ XLR de novo |
| 17/Mito077/Developmental and epileptic encephalopathy 54/M/37yrs^17^ | GDD, language impairment, febrile convulsions, hearing loss, hypotonia, weight loss, headaches, fatigue, dizziness, autism, anxiety, OCD (33yrs) | NR | NR | HTZ c.804-9_804-6del in *HNRPNU*/ ES trio/ AD de novo |
| 18/Mito085/Spinocerebellar ataxia 5/F/71yrs^18^ | Diplopia, myalgia, ataxia with ataxic gait (6yrs) | NR | NR | HTZ c.3437G>A, p.Arg1146Gln in *SPTBN2*/ ES singleton/ AD unknown |
| 19/Mito136/Congenital disorder of glycosylation, type I/F/27yrs^19^ | Dilated cardiomyopathy, myopathy, distal tremor, cleft palate, Pierre Robin syndrome (2yrs) | NR | NR | CMP HTZ c.988G>C, p.Gly330Arg (paternal)/ c.787G>T, p.Asp263Tyr (maternal) in *PGM1*/ ES trio/ AR |
| 20/Mito183/Spastic ataxia 1/M/49yrs^20^ | Dysphagia, cerebellar ataxia, fatty liver, peripheral neuropathy (8yrs) | Brain MRI=T2 hyperintensity in supratentorial WM | NR | HTZ c.340+2T>G in *VAMP1*/ ES singleton/ AD unknown |
| 21/Mito330/Usher syndrome type 2A/F/19yrs^14^ | Sensorineural hearing loss, verbal delay, motor delay, postural hypotension (NB) | Brain MRI=parahippocampal gyri, cerebral and cerebellar volume loss | UOA 3-MGA=16.2 | HMZ c.5776+1G>A in *USH2A*/ ES singleton/ AR unknown |
| 22/Mito384/Developmental and epileptic encephalopathy 64/M/36yrs^22^ | Developmental delay with regression, cognitive dysfunction, epilepsy, spasticity, hypoxic ischemic encephalopathy (1yr) | Brain MRI= cerebral and cerebellar atrophy, ventriculomegaly, gliosis and ischemic change in parietal lobes | Blood lactate=4.3 | HTZ c.1532G>A, p.Arg511Gln in *RHOBTB2*/ ES duo/ AD not maternal |

^1-22^Superscript indicates family number

**Abbreviations**: AD=autosomal dominant; AR=autosomal recessive; ASD=autism spectrum disorder; DM=diabetes mellitus; ECHO=echocardiography; EEG=electroencephalography; EMG=electromyography; GDD=global developmental delay; HEMZ= hemizygous; HMZ=homozygous; HTZ=heterozygous; mo(s)=month(s); MRI=magnetic resonance imaging; NR=not reported; PAA=plasma amino acids; PVWM=periventricular won; RRFs=ragged red fibers; UOA=urine organic acid; ES=exome sequencing; WM=white matter; yr(s)=year(s)

**Reference Ranges:** PAA alanine adult: 180-600 umol/L; PAA alanine child: 13-48 umol/L; blood lactate child: 0.5-2.2 mmol/L; UOA 3-MGA adult: 15-87; UOA lactic acic adult: <400 mmol/mol creatinine; plasma 3-MGA adult=; plasma 3-MGA child= 103-384 nmol/L; CSF lactate adult: 1.1-2.4 mmol/L; CSF alanine: <48 mmol/L

**Supplemental Table 6**. Statistical comparison of phenotypes, biochemical features, muscle biopsy results, and neuroimaging across groups

| **Features** | **Abnormal clinical, biochemical and organ involvement** | **Group 1:**  **mtDNA- PMD**  **(total n=41; adult n=36; children n=5)**  **(percentages%), p value** | **Group 2: nDNA-PMD**  **(total n=30; adult n=13; children n=17), p value** | **Comparison of Group 1 and Group 2**  **Compare total, adults and children** | **Group 3: non-PMD (total n=22; adult n=8; children n=14), p value** | **Comparison of Group 1 and Group 3**  **Compare total, adults and children** | **Comparison of Group 2 and Group 3**  **Compare total, adults and children** | **Group 4: No genetic diagnosis (total n=204; adult n=140; children n=64), p value** | **Comparison of Group 1 and Group 4**  **Compare total, adults and children** | **Comparison of Group 2 and Group 4**  **Compare total, adults and children** |
| --- | --- | --- | --- | --- | --- | --- | --- | --- | --- | --- |
| Phenotypes | Neurodevelopmental phenotype | Total=10 (24)  Adult=6 (17)  Children=4 (8), 0.009023* | Total=16 (53)  Adult=5 (39)  Children=11 (65), 0.2685 | 0.02385*, 0.1327, 1 | Total=17 (77)  Adult=4 (50)  Children=13 (93), 0.03934* | 0.00000004271*, 0.06415, 0.4678 | 0.0005808*, 0.6731, 0.0940 | Total=67 (33)  Adult=33 (24)  Children=34 (53), 0.00005035* | 1.010x10^-18^*, 0.5007, 0.3698 | 5.200x10^-9^*, 0.3114, 0.4259 |
|  | Muscular phenotype | Total=18 (44)  Adult=16 (32)  Children=2 (40), 1 | Total=16 (53)  Adult=9 (69)  Children=8 (47), 0.2828 | 0.3412, 0.1963, 1 | Total=10 (45)  Adult=5 (6)  Children=5 (36), 0.3777 | 0.001117*, 0.4485, 1 | 0.01643*, 1, 0.7168 | Total=94 (46)  Adult=74 (53)  Children=20 (31), 0.004241* | 3.835x10^-15^*, 0.4552, 0.6508 | 8.729x10^-10^*, 0.3840, 0.2584 |
|  | Ophthalmologic phenotype | Total=15 (37)  Adult=14 (39)  Children=1 (20), 0.6357 | Total=6 (20)  Adult=4 (31)  Children=2 (12), 0.3598 | 0.1887, 0.7426, 1 | Total=0 | 1, 0.0410*, 0.2632 | 1, 0.1312, 0.4882 | Total=58 (28)  Adult=51 (36)  Children=7 (11), 0.0001117* | 1.225x10^-13^*, 0.8472, 0.4706 | 2.520x10^-16^*, 0.7713, 1 |
|  | GI phenotype | Total=1 (2)  Adult=1 (3)  Children=0, 1 | Total=4 (13)  Adult=3 (23)  Children=1 (6), 0.2903 | 0.1548, 0.05197, 1 | Total=2 (9)  Adult=1 (13)  Children=1 (7), 1 | 0.003322*, 0.3340, 1 | 0.03024*, 1, 1 | Total=19 (9)  Adult=11 (9)  Children=7 (3), 0.5952 | 2.934x10^-14^*, 0.4637, 1 | 1.001x10^-9^, 0.1007, 1 |
|  | Endocrinological phenotype | Total=6 (15)  Adult=6 (17)  Children=0, 1 | Total=1 (3)  Adult=1 (8)  Children=0, 0.4333 | 0.2260, 0.6577, NA | Total=0 | 1, 0.5728, 1 | 1, 1, NA | Total=18 (9)  Adult=16 (11)  Children=2 (3), 0.06312 | 2.0786x10^-10^*, 0.4024, 1 | 2.599x10^-12^, 1, 1 |
|  | Renal phenotype | Total=2 (5)  Adult=1 (3)  Children=1 (20), 0.2317 | Total=1 (3)  Adult=0  Children=1 (6), 1 | 1, 1, 0.4113 | Total=0 | 1, 1, 0.2632 | 1, NA, 1 | Total=9 (4)  Adult=7 (5)  Children=2 (3), 0.7228 | 0.00000002195*, 1, 0.2048 | 4.719x10^-8^*, 1, 0.5117 |
|  | Liver phenotype | Total=1 (2)  Adult=1 (3)  Children=0, 1 | Total=3 (10)  Adult=1 (8)  Children=2 (12), 1 | 0.3038, 0.4643, 1 | Total=1 (9)  Adult=1 (13)  Children=0, 0.3636 | 0.04762*, 0.3340, NA | 0.1290, 1, 0.4882 | Total=13 (6)  Adult=7 (5)  Children=6 (9), 0.2346 | 1.263x10^-11^*, 1, 1 | 1.531x10^-8^*, 0.5171, 0.6717 |
|  | Cardiac phenotype | Total=4 (10)  Adult=3 (8)  Children=1 (20), 0.4183 | Total=6 (20)  Adult=2 (15)  Children=4 (24), 0.6725 | 0.3042, 0.5984, 1 | Total=3 (14)  Adult=1 (13)  Children=2 (14), 1 | 0.002643*, 0.5661, 1 | 0.01540*, 1, 0.6638 | Total=24 (12)  Adult=15 (11)  Children=9 (14), 0.4903 | 5.153x10^-14^*, 1, 0.5546 | 5.094x10^-10^*, 0.6397, 0.4562 |
|  | Lung phenotype | Total=0 | Total=0 | NA | Total=0 | NA | NA | Total=10 (5)  Adult=6 (4)  Children=4 (6), 0.5089 | 7.826x10^-11^*, 0.3487, 1 | 1.180x10^-9^*, 1, 0.5743 |
|  | Peripheral nervous system phenotype | Total=7 (17)  Adult=7 (20)  Children=0, 0.5668 | Total=5 (17)  Adult=3 (23)  Children=2 (12), 0.6278 | 1, 1, 1 | Total=2 (9)  Adult=1 (13)  Children=1 (7), 1 | 0.03987*, 1, 1 | 0.04234*, 1, 1 | Total=25 (13)  Adult=21 (15)  Children=4 (6), 0.1062 | 3.208x10^-12^*, 0.6091, 1 | 8.871x10^-11^*, 0.4309, 0.6006 |
|  | Movement disorder | Total=7 (17)  Adult=6 (17)  Children=1 (20), 1 | Total=14 (47)  Adult=6 (46)  Children=8 (47), 1 | 0.009180*, 0.05768, 0.3602 | Total=11 (50)  Adult=5 (63)  Children=6 (43), 0.6594 | 0.0000006269*, 0.01566*, 0.6027 | 0.002562*, 0.6594, 1 | Total=38 (19)  Adult=26 (19)  Children=12 (19), 1 | 1.702x10^-15^*, 1, 1 | 9.896x10^-8^*, 0.03029*, 0.02574* |
|  | Stroke and stroke-like episode | Total=7 (17)  Adult=6 (17)  Children=1 (20), 1 | Total=1 (3)  Adult=0  Children=1 (6), 1 | 0.1265, 0.1749, 0.4113 | Total=0 | 1, 0.5728, 0.2632 | 1, NA, 1 | Total=9 (4)  Adult=8 (6)  Children=1 (2), 0.2780 | 0.000004566*, 0.04140*, 0.1407 | 4.719x10^-8^*, 1, 0.3778 |
|  | Hypotonia | Total=1 (2)  Adult=0  Children=1 (20), 0.1220 | Total=6 (20)  Adult=1 (8)  Children=5 (29), 0.1961 | 0.03674*, 0.2653, 1 | Total=9 (41)  Adult=2 (25)  Children=7 (50), 0.3802 | 0.000000003991*, 0.02960*, 0.3378 | 0.00002362*, 0.5308, 0.2883 | Total=19 (9)  Adult=4 (3)  Children=15 (23), 0.00001013* | 9.781x10^-15^*, 0.5829, 1 | 1.165x10^-8^*, 0.3625, 0.7523 |
|  | Headache | Total=11 (27)  Adult=9 (25)  Children=2 (40), 0.5977 | Total=1 (3)  Adult=1 (8)  Children=0, 0.4333 | 0.01013*, 0.2528, 0.04329* | Total=1 (5)  Adult=1 (13)  Children=0, 0.3636 | 0.2857, 0.6585, 0.05848 | 0.06452, 1, NA | Total=37 (18)  Adult=33 (24)  Children=4 (6), 0.002869* | 1.382x10^-12^*, 0.8297, 0.05656 | 3.804x10^-18^*, 0.2995, 0.5743 |
|  | Hearing loss* | Total=14 (34)  Adult=12 (33)  Children=2 (40), 1 | Total=3 (10)  Adult=0  Children=3 (18), 0.2379 | 0.02438*, 0.02146*, 0.5481 | Total=5 (23)  Adult=3 (38)  Children=2 (14), 0.3089 | 0.008483*, 1, 0.2722 | 0.0001725*, 0.04211*, 1 | Total=31 (15)  Adult=27 (19)  Children=4 (6), 0.01959* | 0.000000001359*, 0.07651, 0.05656 | 2.571x10^-14^*, 0.1262, 0.1566 |
|  | Seizure | Total=7 (17)  Adult=5 (14)  Children=2 (40), 0.1955 | Total=4 (13)  Adult=3 (23)  Children=1 (6), 0.2903 | 0.7498, 0.4225, 0.1169 | Total=3 (14)  Adult=0  Children=3 (21), 0.2727 | 0.009061*, 0.5661, 0.5696 | 0.006415*, 0.2571, 0.3041 | Total=34 (17)  Adult=14 (10)  Children=20 (31), 0.0004049* | 9.134x10^-15^*, 0.5479, 0.6508 | 4.556x10^-14^*, 0.1610, 0.05796 |
| Biochemical features | Elevated blood lactate | Total=5 (12)  Adult=3 (8)  Children=2 (40), 0.1039 | Total=7 (23)  Adult=2 (15)  Children=5 (29), 0.4268 | 0.3367, 0.5984, 1 | Total=2 (9)  Adult=1 (13)  Children=1 (7), 1 | 0.02326*, 0.5661, 0.1548 | 0.07258, 1, 0.1848 | Total=15 (7)  Adult=5 (4)  Children=10 (16), 0.006574* | 9.539x10^-10^*, 0.2094, 0.2057 | 0.0000004945*, 0.1099, 0.2891 |
|  | Elevated plasma alanine | Total=1 (2)  Adult=1 (3)  Children=0, 1 | Total=4 (13)  Adult=4 (31)  Children=1 (6), 0.1377 | 0.07634, 0.01417*, 1 | Total=0 | 1, 1, NA | 1, 0.1312, 1 | Total=16 (8)  Adult=8 (6)  Children=8 (13), 0.1574 | 2.936x10^-13^*, 0.6881, 1 | 2.052x10^-8^, 0.01080*, 0.6762 |
|  | Elevated CSF lactate | Total=4 (10)  Adult=4 (11)  Children=0, 1 | Total=2 (7)  Adult=1 (8)  Children=1 (6), 1 | 1, 1, 1 | Total=0 | 1, 1, NA | 1, 1, 1 | Total=1 (1)  Adult=0  Children=1 (2), 0.3137 | 0.1190, 0.001525*, 1 | 0.09677, 0.08497, 0.3778 |
|  | Elevated CSF alanine | Total=1 (2)  Adult=1 (3)  Children=0, 1 | Total=1 (3)  Adult=0  Children=1 (6), 1 | 1, 1, 1 | Total=0 | 1, 1, NA | 1, NA, 1 | Total=1 (1)  Adult=0  Children=1 (2), 0.3137 | 0.04762*, 0.2045, 1 | 0.06452, NA, 0.3778 |
|  | Elevated urine lactic acid | Total=2 (5)  Adult=2 (6)  Children=0 | Total=1 (3)  Adult=0  Children=1 (6), 1 | 1, 1, 1 | Total=0 | 1, 1, NA | 1, NA, 1 | Total=6 (3)  Adult=1 (1)  Children=5 (8), 0.01229* | 0.000002608*, 0.1067, 1 | 0.000003594*, 1, 1 |
|  | Elevated urine 3-methylglutaconic acid | Total=0 | Total=4 (13)  Adult=1 (8)  Children=3 (18), 0.6129 | 0.02821*, 0.2653, 1 | Total=2 (9)  Adult=1 (13)  Children=1 (7), 1 | 0.001107*, 0.1818, 1 | 0.03024*, 1, 0.6067 | Total=1 (1)  Adult=1 (1)  Children=0, 1 | 0.02381*, 1, NA | 0.1613, 0.1632, 0.007970* |
|  | Abnormal urine Krebs cycle intermediates | Total=1 (2)  Adult=1 (3)  Children=0, 1 | Total=1 (3)  Adult=0  Children=1 (6), 1 | 1, 1, 1 | Total=1 (5)  Adult=1 (13  Children=0, 0.3636 | 0.04762*, 0.3340, NA | 0.06451, 0.3810, 1 | Total=2 (1)  Adult=0  Children=2 (3), 0.09736 | 0.003322*, 0.2045, 1 | 0.006048*, NA, 0.5117 |
| Muscle biopsy ETC | Complex I | Total=3 (7)  Adult=2 (6)  Children=1 (20), 0.3302 | Total=1 (3)  Adult=1 (8)  Children=0, 0.4333 | 0.6329, 1, 0.2273 | Total=0 | 1, 1, 0.2632 | 1, 1, NA | Total=3 (2)  Adult=0  Children=3 (5), 0.02988* | 0.001510*, 0.04091*, 0.2650 | 0.0007331*, 0.08497, 1 |
|  | Complex I+III/CS | Total=1 (2)  Adult=1 (3)  Children=0, 1 | Total=0 | 1, 1, NA | Total=0 | 1, 1, NA | NA | Total=0 | 1, 0.2045, NA | NA |
|  | Complex I+III/II+III | Total=1 (2)  Adult=1 (3)  Children=0, 1 | Total=0 | 1, 1, NA | Total=0 | 1, 1, NA | NA | Total=0 | 1, 0.2045, NA | NA |
|  | Complex II | Total=0 | Total=0 | NA | Total=1 (5)  Adult=1 (13)  Children=0, 0.3636 | 0.02381*, 0.1818, NA | 0.03226*, 0.3810, NA | Total=2 (1)  Adult=0  Children=2 (3), 0.09736 | 0.001107*, NA, 1 | 0.002016*, NA, 1 |
|  | Complex III | Total=1 (2)  Adult=1 (3)  Children=0, 1 | Total=0 | 1, 1, NA | Total=0 | 1, 1, NA | NA | Total=1 (1)  Adult=1 (1)  Children=0, 1 | 0.04762*, 0.3682, NA | 0.03226*, 1, NA |
|  | Complex IV | Total=1 (2)  Adult=1 (3)  Children=0, 1 | Total=1 (3)  Adult=1 (8)  Children=0, 0.4333 | 1, 0.4643, NA | Total=0 | 1, 1, NA | 1, 1, NA | Total=5 (2)  Adult=2 (1)  Children=3 (5), 0.1791 | 0.000004377*, 0.4989, 1 | 0.00001848*, 0.2353, 1 |
|  | Complex IV/CS | Total=0 | Total=0 | NA | Total=1 (5)  Adult=1 (13)  Children=0, 0.3636 | 0.02381*, 0.1818, NA | 0.03226*, 0.3810, NA | Total=1 (1)  Adult=1 (1)  Children=0, 1 | 0.02381*, 1, NA | 0.03226*, 1, NA |
|  | Complex V | Total=1 (2)  Adult=1 (3)  Children=0, 1 | Total=0 | 1, 1, NA | Total=0 | 1, 1, NA | NA | Total=1 (1)  Adult=1 (1)  Children=0, 1 | 0.04762*, 0.3682, NA | 0.03226*, 1, NA |
|  | Complex V/CS | Total=0 | Total=0 | NA | Total=0 | NA | NA | Total=1 (1)  Adult=1 (1)  Children=0, 1 | 0.02381*, 1, NA | 0.03226*, 1, NA |
| Muscle biopsy electron microscopy | Enlarged mitochondria | Total=1 (2)  Adult=1 (3)  Children=0, 1 | Total=0 | 1, 1, NA | Total=1 (5)  Adult=1 (13)  Children=0, 0.3636 | 0.04762*, 0.3340, NA | 0.03226*, 0.3810, NA | Total=1 (1)  Adult=1 (1)  Children=0, 1 | 0.04762*, 0.3682, NA | 0.03226*, 1, NA |
|  | Accumulation of mitochondria | Total=3 (7)  Adult=2 (6)  Children=1 (20), 0.3302 | Total=0 | 0.2575, 1, 0.2273 | Total=1 (5)  Adult=1 (13)  Children=0, 0.3636 | 0.09524, 0.4609, 0.2632 | 0.03226*, 0.3810, NA | Total=12 (6)  Adult=10 (7)  Children=2 (3), 0.3472 | 1.706x10^-9^*, 1, 0.2048 | 9.043x10^-11^*, 1, 1 |
|  | Other/unclassified mitochondrial abnormality | Total=6 (15)  Adult=6 (17)  Children=0, 1 | Total=2 (7)  Adult=1 (8)  Children=1 (6), 1 | 0.4532, 0.6577, 1 | Total=0 | 1, 0.5728, NA | 1, 1, 1 | Total=3 (1)  Adult=2 ()  Children=1 (2), 1 | 0.006342*, 0.001037*, 1 | 0.001833*, 0.2353, 0.3778 |
| Muscle biopsy muscle histopathology | Ragged red fibers | Total=10 (24)  Adult=10 (28)  Children=0, 0.3102 | Total=2 (7)  Adult=1 (8)  Children=1 (6), 1 | 0.06013, 0.2461, 1 | Total=1 (5)  Adult=1 (13)  Children=0, 0.3636 | 0.2619, 0.6563, NA | 0.09677, 1, 1 | Total=12 (6)  Adult=12 (9)  Children=0, 0.01983* | 0.000002424*, 0.004112*, NA | 8.229x10^-9^*, 1, 0.2099 |
|  | Ragged blue fibers | Total=0 | Total=0 | NA | Total=0 | NA | NA | Total=3 (1)  Adult=3 (2)  Children=0, 0.5534 | 0.00007551*, 1, NA | 0.0001833*, 1, NA |
|  | SDH staining | Total=1 (2)  Adult=1 (3)  Children=0, 1 | Total=2 (7)  Adult=2 (15)  Children=0, 0.1793 | 0.5696, 0.1679, NA | Total=2 (9)  Adult=1 (13)  Children=1 (7), 1 | 0.003322*, 0.3340, 1 | 0.01210*, 1, 0.4516 | Total=9 (4)  Adult=7 (5)  Children=2 (3), 0.7228 | 0.000000003991*, 1, 1 | 0.0000002595*, 0.1708, 1 |
|  | COX staining | Total=7 (17)  Adult=7 (19)  Children=0, 0.5668 | Total=3 (10)  Adult=2 (15)  Children=1 (6), 0.5645 | 0.5018, 1, 1 | Total=3 (14)  Adult=1 (13)  Children=2 (14), 1 | 0.009061*, 1, 1 | 0.003666*, 1, 0.5764 | Total=24 (12)  Adult=20 (1)  Children=4 (6), 0.1079 | 6.617x10^-12^*, 0.4431, 1 | 4.171x10^-12^*, 1, 1 |
| Neuroimaging | Abnormal Brain MRI | Total=18 (44)  Adult=15 (42)  Children=3 (60), 0.6384 | Total=14 (47)  Adult=4 (31)  Children=11 (59), 0.1394 | 0.6379, 0.7408, 1 | Total=9 (41)  Adult=3 (38)  Children=6 (43), 1 | 0.002197*, 1, 0.6285 | 0.006922*, 1, 0.2895 | Total=84 (41)  Adult=52 (37)  Children=32 (50), 0.09319 | 2.668x10^-14^*, 0.7011, 1 | 7.443x10^-11^*, 0.7693, 0.4129 |
|  | Abnormal Brain MRS | Total=1 (2)  Adult=1 (3)  Children=0, 1 | Total=2 (7)  Adult=0  Children=2 (12), 0.4920 | 0.5696, 1, 1 | Total=1 (5)  Adult=0  Children=1 (7), 1 | 0.04762*, 1, 1 | 0.09677, NA, 1 | Total=2 (1)  Adult=0  Children=2 (3), 0.09736 | 0.003322*, 0.2045, 1 | 0.01210*, NA, 0.1924 |
| EEG | Abnormal EEG | Total=3 (7)  Adult=3 (8)  Children=0, 1 | Total=1 (3)  Adult=0  Children=1 (6), 1 | 0.6329, 0.5555, 1 | Total=2 (9)  Adult=0  Children=2 (14), 0.5152 | 0.01107*, 1, 1 | 0.006048*, NA, 0.5764 | Total=15 (8)  Adult=4 (3)  Children=11 (18), 0.0006533* | 5.021x10^-11^*, 0.1520, 0.5847 | 4.639x10^-11^*, 1, 0.4439 |
| Neurophysiological studies | Abnormal EMG | Total=3 (7)  Adult=3 (8)  Children=0, 1 | Total=0 | 0.2575, 0.5555, NA | Total=1 (5)  Adult=1 (13)  Children=0, 0.3636 | 0.09524, 0.5661, NA | 0.03226*, 0.3810, NA | Total=13 (6)  Adult=12 (9)  Children=1 (2), 0.06707 | 5.053x10^-10^*, 1, 1 | 2.734x10^-11^*, 0.6006, 1 |
|  | Abnormal NCV | Total=1 (2)  Adult=1 (3)  Children=0, 1 | Total=0 | 1, 1, NA | Total=0 | 1, 1, NA | NA | Total=4 (2)  Adult=4 (3)  Children=0, 0.3111 | 0.00003356*, 1, NA | 0.00002156*, 1, NA |

*Most likely sensorineural hearing loss, but in some patients, this was not specified.

*Significance at alpha=0.05

**Phenotypes:** Cardiac phenotype=Cardiomyopathy (dilated, hypertrophic), arrythmia, cardiac arrest, heart failure; Endocrinological phenotype=Diabetes, thyroid and parathyroid impairment; Gastrointestinal (GI) phenotype=constipation, diarrhea, vomiting, gastroesophageal reflux, gastroesophageal dysmotility; Hypotonia=undifferentiated peripheral and central; Liver phenotype=Hepatic steatosis, cholestasis, chronic liver disease; Lung phenotype=Respiratory insufficiency, pulmonary restrictive lung disease, pulmonary fibrosis, alveolar proteinosis; Movement disorders=Ataxia, dyskinesia, chorea, chorea athetosis, tremor, dystonia, myo-clonus, hyperkinesia, bradykinesia; Muscular phenotype=muscle weakness, muscle pain, rhabdomyolysis, exercise intolerance; Neurodevelopmental phenotype=developmental delay, intellectual disability, cognitive dysfunction, behavioral disorders; Ophthalmologic phenotype=retinopathy, external ophthalmoplegia, ptosis, vision impairments; Peripheral nervous system phenotype= neuropathy, peripheral tingling and pin-pricks; Renal phenotype=Acute and chronic kidney insufficiency, renal tubular acidosis, tubulopathy, Fanconi syndrome, renal transplant; Stroke and stroke-like episodes=neurological signs of stroke and confirmation with MRI or without MRI

**Supplemental Table 7.** Statistical comparison of all PMD (Groups 1+2) compared with non-PMD (Group 3) for phenotypes, biochemical features, muscle biopsy results, and neuroimaging.

| **Features** | **Abnormal clinical, biochemical and organ involvement** | **All PMD**  **total n=71 adult n=49 children n=22**  **(percentages%), Comparison Adult vs Children** | **Non-PMD (total n=22; adult n=8; children n=14), Comparison Adult vs Children** | **Comparison of All PMD and and Other genetic diagnosis**  **Compare total, adults and children** |
| --- | --- | --- | --- | --- |
| Phenotypes | Neurodevelopmental phenotype | Total=26 (37)  Adult=11 (22)  Children=15 (68), 0.0004021* | Total=17 (77)  Adult=4 (50)  Children=13 (93), 0.03934* | 0.0000006869*, 0.1875, 0.1150 |
|  | Muscular phenotype | Total=34 (48)  Adult=25 (51)  Children=10 (45), 0.7985 | Total=10 (45)  Adult=5 (6)  Children=5 (36), 0.3777 | 0.001834*, 0.7089, 0.7317 |
|  | Ophthalmologic phenotype | Total=21 (30)  Adult=18 (37)  Children=3 (14), 0.0552 | Total=0 | 1, 0.04634*, 0.2667 |
|  | GI phenotype | Total=5 (7)  Adult=4 (8)  Children=1 (5), 1 | Total=2 (9)  Adult=1 (13)  Children=1 (7), 1 | 0.007991*, 0.5446, 1 |
|  | Endocrinological phenotype | Total=7 (10)  Adult=7 (14)  Children=0, 0.09076 | Total=0 | 1, 0.5769, 1 |
|  | Renal phenotype | Total=3 (4)  Adult=1 (2)  Children=2 (9), 0.2250 | Total=0 | 1, 1, 0.5111 |
|  | Liver phenotype | Total=4 (6)  Adult=2 (4)  Children=2 (9), 0.5828 | Total=1 (9)  Adult=1 (13)  Children=0, 0.3636 | 0.06944, 0.3703, 0.5111 |
|  | Cardiac phenotype | Total=10 (14)  Adult=5 (12)  Children=5 (23), 0.2663 | Total=3 (14)  Adult=1 (13)  Children=2 (14), 1 | 0.004412*, 1, 0.6810 |
|  | Lung phenotype | Total=0 | Total=0 | 1, 1, 1 |
|  | Peripheral nervous system phenotype | Total=12 (17)  Adult=10 (20)  Children=2 (9), 0.3185 | Total=2 (9)  Adult=1 (13)  Children=1 (7), 1 | 0.03463*, 1, 1 |
|  | Movement disorder | Total=21 (30)  Adult=12 (24)  Children=9 (41), 0.1734 | Total=11 (50)  Adult=5 (63)  Children=6 (43), 0.6594 | 0.000009214*, 0.04333*, 1 |
|  | Stroke and stroke-like episode | Total=8 (11)  Adult=6 (12)  Children=2 (9), 1 | Total=0 | 1, 0.5796, 1 |
|  | Hypotonia | Total=7 (10)  Adult=1 (20)  Children=6 (27), 0.002877* | Total=9 (41)  Adult=2 (25)  Children=7 (50), 0.3802 | 0.00000004933*, 0.04880*, 0.2859 |
|  | Headache | Total=12 (17)  Adult=10 (14)  Children=2 (9), 0.3185 | Total=1 (5)  Adult=1 (13)  Children=0, 0.3636 | 0.1806, 1, 0.5111 |
|  | Hearing loss* | Total=17 (24)  Adult=12 (24)  Children=5 (23), 1 | Total=5 (23)  Adult=3 (38)  Children=2 (14), 0.3089 | 0.001425*, 0.4218, 0.6810 |
|  | Seizure | Total=11 (15)  Adult=8 (16)  Children=3 (14), 1 | Total=3 (14)  Adult=0  Children=3 (21), 0.2727 | 0.005615*, 0.5841, 0.6582 |
| Biochemical features | Elevated blood lactate | Total=12 (17)  Adult=5 (10)  Children=7 (32), 0.03868* | Total=2 (9)  Adult=1 (13)  Children=1 (7), 1 | 0.03463*, 1, 0.1150 |
|  | Elevated plasma alanine | Total=5 (7)  Adult=5 (10)  Children=1 (5), 0.6583 | Total=0 | 1, 1, 1 |
|  | Elevated CSF lactate | Total=6 (8)  Adult=5 (10)  Children=1 (5), 0.6583 | Total=0 | 1, 1, 1 |
|  | Elevated CSF alanine | Total=2 (3)  Adult=1 (2)  Children=1 (5), 0.5268 | Total=0 | 1, 1, 1 |
|  | Elevated urine lactic acid | Total=3 (4)  Adult=2 (4)  Children=1 (5), 1 | Total=0 | 1, 1, 1 |
|  | Elevated urine 3-methylglutaconic acid | Total=4 (6)  Adult=1 (2)  Children=3, 0.08519 | Total=2 (9)  Adult=1 (13)  Children=1 (7), 1 | 0.005708*, 0.2632, 1 |
|  | Abnormal urine Krebs cycle intermediates | Total=2 (3)  Adult=1 (2)  Children=1 (5), 0.5268 | Total=1 (5)  Adult=1 (13  Children=0, 0.3636 | 0.04167*, 0.2632, 1 |
| Muscle biopsy ETC | Complex I | Total=4 (6)  Adult=3 (6)  Children=1 (5), 1 | Total=0 | 1, 1, 1 |
|  | Complex I+III/CS | Total=1 (1)  Adult=1 (2)  Children=0, 1 | Total=0 | 1, 1, 1 |
|  | Complex I+III/II+III | Total=1 (1)  Adult=1 (2)  Children=0, 1 | Total=0 | 1, 1, 1 |
|  | Complex II | Total=0 | Total=1 (5)  Adult=1 (13)  Children=0, 0.3636 | 0.01389*, 0.1404, 1 |
|  | Complex III | Total=1 (1)  Adult=1 (2)  Children=0, 1 | Total=0 | 1, 1, 1 |
|  | Complex IV | Total=1 (1)  Adult=1 (2)  Children=0, 1 | Total=0 | 1, 1, 1 |
|  | Complex IV/CS | Total=0 | Total=1 (5)  Adult=1 (13)  Children=0, 0.3636 | 0.01389*, 0.1404, 1 |
|  | Complex V | Total=1 (1)  Adult=1 (2)  Children=0, 1 | Total=0 | 1, 1, 1 |
|  | Complex V/CS | Total=0 | Total=0 | 1, 1, 1 |
| Muscle biopsy electron microscopy | Enlarged mitochondria | Total=1 (1)  Adult=1 (2)  Children=0, 1 | Total=1 (5)  Adult=1 (13)  Children=0, 0.3636 | 0.02778*, 0.2632, 1 |
|  | Accumulation of mitochondria | Total=3 (4)  Adult=2 (4)  Children=1 (5), 1 | Total=1 (5)  Adult=1 (13)  Children=0, 0.3636 | 0.05556, 0.3703, 1 |
|  | Other/unclassified mitochondrial abnormality | Total=8 (11)  Adult=7 (14)  Children=1 (5), 0.4204 | Total=0 | 1, 0.5769, 1 |
| Muscle biopsy muscle histopathology | Ragged red fibers | Total=12 (17)  Adult=11 (22)  Children=1 (5), 0.08874 | Total=1 (5)  Adult=1 (13)  Children=0, 0.3636 | 0.1806, 1, 1 |
|  | Ragged blue fibers | Total=0 | Total=0 | 1, 1, 1 |
|  | SDH staining | Total=3 (4)  Adult=3 (6)  Children=0, 0.5473 | Total=2 (9)  Adult=1 (13)  Children=1 (7), 1 | 0.003805*, 0.4636, 0.3889 |
|  | COX staining | Total=10 (14)  Adult=9 (18)  Children=1 (5), 0.1576 | Total=3 (14)  Adult=1 (13)  Children=2 (14), 1 | 0.004412*, 1, 0.5471 |
| Neuroimaging | Abnormal Brain MRI | Total=32 (45)  Adult=19 (39)  Children=14 (64), 0.07241 | Total=9 (41)  Adult=3 (38)  Children=6 (43), 1 | 0.002626*, 1, 0.3074 |
|  | Abnormal Brain MRS | Total=3 (4)  Adult=1 (2)  Children=2 (9), 0.2250 | Total=1 (5)  Adult=0  Children=1 (7), 1 | 0.05556, 1, 1 |
| EEG | Abnormal EEG | Total=4 (6)  Adult=3 (6)  Children=1 (5), 1 | Total=2 (9)  Adult=0  Children=2 (14), 0.5151 | 0.005708*, 1, 0.5471 |
| Neurophysiological studies | Abnormal EMG | Total=3 (4)  Adult=3 (6)  Children=0, 0.5473 | Total=1 (5)  Adult=1 (13)  Children=0, 0.3636 | 0.05556, 0.4636, 1 |
|  | Abnormal NCV | Total=1 (1)  Adult=1 (2)  Children=0, 1 | Total=0 | 1, 1, 1 |

*Most likely sensorineural hearing loss, but in some patients, this was not specified.

*Significance at alpha=0.05

**Phenotypes:** Cardiac phenotype=Cardiomyopathy (dilated, hypertrophic), arrythmia, cardiac arrest, heart failure; Endocrinological phenotype=Diabetes, thyroid and parathyroid impairment; Gastrointestinal (GI) phenotype=constipation, diarrhea, vomiting, gastroesophageal reflux, gastroesophageal dysmotility; Hypotonia=undifferentiated peripheral and central; Liver phenotype=Hepatic steatosis, cholestasis, chronic liver disease; Lung phenotype=Respiratory insufficiency, pulmonary restrictive lung disease, pulmonary fibrosis, alveolar proteinosis; Movement disorders=Ataxia, dyskinesia, chorea, chorea athetosis, tremor, dystonia, myo-clonus, hyperkinesia, bradykinesia; Muscular phenotype=muscle weakness, muscle pain, rhabdomyolysis, exercise intolerance; Neurodevelopmental phenotype=developmental delay, intellectual disability, cognitive dysfunction, behavioral disorders; Ophthalmologic phenotype=retinopathy, external ophthalmoplegia, ptosis, vision impairments; Peripheral nervous system phenotype= neuropathy, peripheral tingling and pin-pricks; Renal phenotype=Acute and chronic kidney insufficiency, renal tubular acidosis, tubulopathy, Fanconi syndrome, renal transplant; Stroke and stroke-like episodes=neurological signs of stroke and confirmation with MRI or without MRI

**Supplemental Table 8**. Predicted protein-protein interactions causing mitochondrial dysfunction in individuals with non-PMD [45,107-115].

| **Study ID/ diagnosis gene** | **Gene thought to cause mitochondrial dysfunction** | **Gene function** | **Gene interaction/confidence score** | **Abnormal investigations** |
| --- | --- | --- | --- | --- |
| Mito044/*MECP2* [107] | *SLC25A4* | Encodes the mitochondrial ADP/ATP, or adenine nucleotide, translocator, which is a homodimer of 30-kD subunits embedded in the mitochondrial inner membrane. The dimer forms a gated pore through which ADP is moved across the inner membrane into the mitochondrial matrix and ATP is moved from the matrix into the cytoplasm | Co-mentioned in scientific literature/NR | Blood lactate=3.1 |
| Mito047/*ATP1A3* | *SLC25A22* | Mitochondrial glutamate carrier, responsible for the transport of glutamate from the cytosol into the mitochondrial matrix with the concomitant import of a proton | Experimentally identified protein-protein binding, detected by affinity chromatography technology assay (BioGRID)/0.63 [108] | Muscle biopsy=complex II deficiency |
| Mito052/*MEF2C* | *MRPL38* | Mitochondrial ribosomal protein L38; Belongs to the phosphatidylethanolamine-binding protein family. Mitochondrion-specific ribosomal protein mL38 subfamily. | No direct evidence, but predicted interaction based on putative homologs co-expressed and found interacting in other organisms (BioGRID)/0.255 | Muscle biopsy=accumulation of mitochondria, large mitochondria |
| Mito066/*COL12A1* | *PTRH2*  *MRPL27*  *MRPL34*  *MRPL3*  *MRPL17*  *MRPL33*  *MRPL16*  *MRPL13*  *MRPL4*  *MRPL46*  *MRPL43* | PTRH2: Peptidyl-tRNA hydrolase 2, mitochondrial; The natural substrate for this enzyme may be peptidyl-tRNAs which drop off the ribosome during protein synthesis.  MRPLs: Mitochondrial ribosomal proteins | Protein-protein interaction (BioGRID)  Detected by proximity labelling technology assay. Protein-protein interaction,  detected by proximity-dependent biotin identification assay (IntAct)/  (0.263) | Muscle biopsy= decreased COX-staining, accumulation of mitochondria |
| Mito070/*MAOA* | *MIEF1*  *HIBADH* | MIEF1: Mitochondrial dynamics protein MID51; Mitochondrial outer membrane protein which regulates mitochondrial fission. Promotes the recruitment and association of the fission mediator dynamin-related protein 1 (DNM1L) to the mitochondrial surface independently of the mitochondrial fission FIS1 and MFF proteins.  HIBADH: 3-hydroxyisobutyrate dehydrogenase, mitochondrial | MIEF1: Experimentally shown to bind or be in same protein complex by  anti-tag coimmunoprecipitation assay and affinity chromatography technology assay (BioGRID)/0.491 [109]  Co-expression (GEO microarray expression data)/0.338 |  |
| Mito077/*HNRNPU* | *MRPS34* | Mitochondrial ribosomal protein Required for mitochondrial translation | Co-expressed and experimentally shown to bind or be in the same protein complex by anti-tag coimmunoprecipitation assay (BioGRID)/0.82 [110,111] | NR |
| Mito136/*PGM1* | *ALDH1B1* | Aldehyde dehydrogenase X, mitochondrial; ALDHs play a major role in the detoxification of alcohol- derived acetaldehyde. They are involved in the metabolism of corticosteroids, biogenic amines, neurotransmitters, and lipid peroxidation. | Co-expression and experimentally shown to bind or be in same protein complex by anti-tag coimmunoprecipitation assay (IntAct)/0.403 | NR |
| Mito154/*ANDKRD11* | GPS2 | G protein pathway suppressor 2; Key regulator of inflammation, lipid metabolism and mitochondrion homeostasis that acts by inhibiting the activity of the ubiquitin-conjugating enzyme UBE2N/Ubc13, thereby inhibiting 'Lys-63'- linked ubiquitination. In the nucleus, can both acts as a corepressor and coactivator of transcription. | Experimentally shown to bind or be in the same protein complex by affinity chromatography technology assay (BioGRID)/0.59 [108] | NR |
| Mito183/*VAMP1* | *STX17* | Syntaxin-17; SNAREs, soluble N-ethylmaleimide-sensitive factor-attachment protein receptors, are essential proteins for fusion of cellular membranes. STX17 is a SNARE of the autophagosome involved in autophagy through the direct control of autophagosome membrane fusion with the lysosome membrane. Involved in mitochondrial dynamics and surveillance via role in fission, organelle contact sites, and autophagy | Co-expression (derived from GEO microarray expression data) and co-mentioned in PubMed abstracts/0.731 [112] | Brain MRI=T2 hyperintensity in supratentorial WM |
| Mito264/*DDX3X* | *ATP5F1B* | ATP synthase subunit beta, mitochondrial; Mitochondrial membrane ATP synthase F(1)F(0) ATP synthase or Complex V) produces ATP from ADP in the presence of a proton gradient across the membrane which is generated by electron transport complexes of the respiratory chain. | Co-expressed (BioGRID)/0.583 | Brain MRI= cerebral parenchymal volume loss, thinning of PVWM/UOA 3-MGA=9.5/ blood lactate=2.5 |
| Mito267/*CACNA1A* [45] | *NDUFB8* | Accessory subunit of the mitochondrial membrane respiratory chain NADH dehydrogenase (Complex I) | Experimental evidence: Bait protein expressed as a DNA binding domain (DBD) fusion and prey expressed as a transcriptional activation domain (TAD) fusion and interaction measured by reporter gene activation (BioGRID)/0.63 [113] | Brain MRI=enlarged lateral ventricles, prominent cerebral sulci, thin corpus callosum, periventricular T2 hyperintensity, stable scaphocephaly |
| Mito299/*PRUNE1* | *NME3* | Nucleoside diphosphate kinase 3; Major role in the synthesis of nucleoside triphosphates other than ATP. The ATP gamma phosphate is transferred to the NDP beta phosphate via a ping-pong mechanism, using a phosphorylated active-site intermediate. Probably has a role in normal hematopoiesis by inhibition of granulocyte differentiation and induction of apoptosis | Co-expressed/0.342 [114] | NR |
| Mito300/*MAST1* | *ERAL1* | ERAL1: GTPase Era, mitochondrial; Probable GTPase that plays a role in the mitochondrial ribosomal small subunit assembly | Co-expression and experimentally shown to bind or be in same protein complex by pull down assay (IntAct)/0.164 | Brain MRI= PVWM volume loss/ Brain MRS=lactate peak |
| Mito384/*RHOBTB2* | *SLC25A1* | Tricarboxylate transport protein, mitochondrial; Citrate transporter that mediates the exchange of mitochondrial citrate for cytosolic malate. Also able to mediate the exchange of citrate for isocitrate, phosphoenolpyruvate, cis- but not trans-aconitate and to a lesser extend maleate and succinate. Important for the bioenergetics of hepatic cells as it provides a carbon source for fatty acid and sterol biosynthesis, and NAD(+) for the glycolytic pathway | Co-expression and experimentally shown to bind or be in same protein complex by affinity chromatography technology assay (BioGRID)/0.171 | Blood lactate=4.3 |
| Mito330/*USH2A* | *ATP5IF1* | ATPase inhibitor, mitochondrial; Endogenous F(1)F(o)-ATPase inhibitor limiting ATP depletion when the mitochondrial membrane potential falls below a threshold and the F(1)F(o)-ATP synthase starts hydrolyzing ATP to pump protons out of the mitochondrial matrix. Required to avoid the consumption of cellular ATP when the F(1)F(o)-ATP synthase enzyme acts as an ATP hydrolase | No experimental evidence but putative homologs were found interacting in other organisms, genes are co-mentioned in PubMed abstracts [115] | UOA 3-MGA=16.2/ brain MRI=parahippocampal gyri, cerebral and cerebellar volume loss |
| Mito389/*USH2A* | *ATP5IF1* | ATPase inhibitor, mitochondrial; Endogenous F(1)F(o)-ATPase inhibitor limiting ATP depletion when the mitochondrial membrane potential falls below a threshold and the F(1)F(o)-ATP synthase starts hydrolyzing ATP to pump protons out of the mitochondrial matrix. Required to avoid the consumption of cellular ATP when the F(1)F(o)-ATP synthase enzyme acts as an ATP hydrolase | No experimental evidence but putative homologs were found interacting in other organisms, genes are co-mentioned in PubMed abstracts/0.161[115] | NR |
